# Calculation of Protein Folding Thermodynamics Using Molecular Dynamics Simulations

**DOI:** 10.1021/acs.jcim.3c01107

**Published:** 2023-11-13

**Authors:** Juan J. Galano-Frutos, Francho Nerín-Fonz, Javier Sancho

**Affiliations:** † Department of Biochemistry, Molecular and Cell Biology, Faculty of Science, University of Zaragoza, 50009 Zaragoza, Spain; ‡ Biocomputation and Complex Systems Physics Institute (BIFI), Joint Unit GBs-CSIC, University of Zaragoza, 50018 Zaragoza, Spain; § Aragon Health Research Institute (IIS Aragón), 50009 Zaragoza, Spain

## Abstract

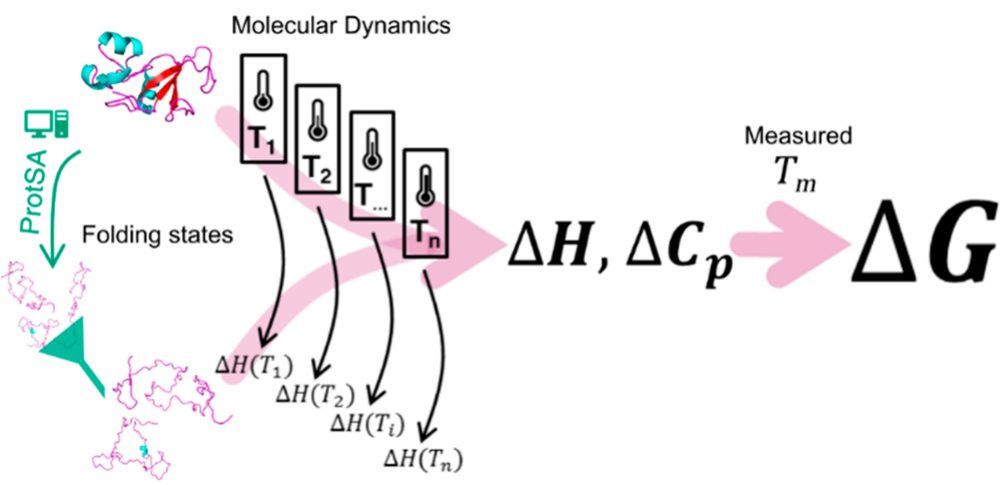

Despite advances in artificial intelligence methods, protein folding remains in many ways an enigma to be solved. Accurate computation of protein folding energetics could help drive fields such as protein and drug design and genetic interpretation. However, the challenge of calculating the state functions governing protein folding from first-principles remains unaddressed. We present here a simple approach that allows us to accurately calculate the energetics of protein folding. It is based on computing the energy of the folded and unfolded states at different temperatures using molecular dynamics simulations. From this, two essential quantities (Δ*H* and ΔCp) are obtained and used to calculate the conformational stability of the protein (Δ*G*). With this approach, we have successfully calculated the energetics of two- and three-state proteins, representatives of the major structural classes, as well as small stability differences (ΔΔ*G*) due to changes in solution conditions or variations in an amino acid residue.

## 1 Introduction

Proteins are very versatile biological molecules,^1^ and thermodynamics can greatly help to understand how they fold and perform useful tasks.^2,3^ Molecular Dynamics (MD) simulation has become a powerful tool to study protein folding and other related processes.^4−12^ However, despite great efforts in developing algorithms and methods to enable longer and better sampled simulations and in improving the accuracy of force fields and water models, significant challenges remain.^13^ On one hand, simulating the protein folding time (from microseconds up to tens of seconds) in explicit solvent remains inaccessible, except for small fast-folding proteins.^5,8,10,11^ On the other hand, work on improving the accuracy of MD force fields seems to have focused on reproducing structural, dynamic, and mechanistic aspects of protein behavior^14−17^ and paid less attention to try to reproduce protein potential energy. One reason for this is the difficulty of obtaining accurate structural models of unfolded ensembles, which has prevented comprehensive studies of this side of the problem, making fine-tuning of the force field parameters challenging. The experimental limitations inherent in quantifying individual atomic interactions and the massive cancellation of interactions that takes place in a protein folding reaction^18^ add to the complexity of the goal.^2^ All of the above has perhaps frustrated the interest of scientists in the use of MD simulations to quantitatively study protein thermodynamics, hindering progress in many applied fields, such as protein design,^19^ drug design,^20^ genetic interpretation,^21^ protein engineering,^22^ or cell engineering.^23^

Recently, we addressed this issue by carrying out accurate, quantitative calculations of conformational stability on two two-state model proteins (barnase and nuclease) through an all-atom MD simulation approach.^24^ The approach circumvents the simulation of the whole folding/unfolding time and is based on separately simulating the two relevant conformations. The folded state is modeled starting from an experimentally determined structure that is conveniently solvated and sampled conformationally. The unfolded state is modeled and sampled from an ensemble of completely unfolded conformations generated by the ProtSA server^25^ that are similarly solvated. From the simulations, the enthalpy change of unfolding (Δ*H*_unf_) is calculated by the difference (unfolded state minus folded state enthalpy averages), while the heat capacity change at constant pressure (ΔC_punf_) is obtained from the temperature dependence of the calculated enthalpy change. As a final step, the calculated thermodynamic quantities (Δ*H*_unf_ and ΔCp_unf_) are combined with the experimentally determined melting temperature (*T*_m_) to calculate the conformational stability of the protein (Δ*G*_unf_) as a function of temperature by means of the Gibbs–Helmholtz equation.^26^

One initial goal of the approach was testing the ability of classical force fields, e.g. Charmm22-CMAP^15^ and AmberSB99-ILDN^16^ (or the more recently released AmberSB99-disp^14^), to yield accurate folding energetics by difference, using systems solvated with explicit water. Thus, the indicated force fields were combined with seven explicit water models, Tip3p,^27^ Tip4p,^27^ Tip4p-d,^28^ Tip4-d-mod,^14^ Tip5p,^29^ Spc,^30^ and Spc/E.^31^ Results obtained from short MD simulations (2 ns productive trajectories per replica) and the combinations of either Charmm22-CMAP or AmberSB99-ILDN with Tip3p allowed, for the two proteins indicated, to finely capture the energy balance between the numerous interactions established between protein and solvent atoms in both the native state and the unfolded ensemble.^32^

In this work, we generalize the described methodology using the most accurate combination of force field and water model found^24^ and a larger conformational sampling (see Methods) and demonstrate the precise correspondence of the thermodynamic quantities calculated on a set of two-state, three-state, apo, holo, wild-type (WT), or mutated proteins with their experimentally determined values. In addition to barnase^33,34^ and nuclease^35,36^ (that are here calculated anew with higher precision^24^), we present the calculation for additional two-state proteins: barley chymotrypsin inhibitor 2 (CI2, truncated variant)^37,38^ and phage T4 lysozyme^39^ (WT and pseudo-WT variant), for a three-state protein: apoflavodoxin from *Anabaena PCC 7119*(40−43) (for which the energetics involved in the two unfolding transitions, F-to-I and I-to-U, is obtained), and for a holoprotein: flavodoxin from *Anabaena PCC 7119* (which contains a flavin mononucleotide (FMN) cofactor, noncovalently bound). Furthermore, we evaluate the capability and limits of the approach to capture small stability changes or small differences between similar systems, e.g. those associated with mutation (ΔΔ*H*_mut-nat_ and ΔΔ*G*_mut-nat_), changes in pH (ΔΔ*H*_pH1-pH2_ and ΔΔ*G*_pH1-pH2_), or individual steps within a multistate unfolding (Δ*H*_unf(F-to-I)_, ΔCp_unf(F-to-I)_, and Δ*G*_unf(F-to-I)_ or Δ*H*_unf(I-to-U)_, ΔCp_unf(I-to-U)_, and Δ*G*_unf(I-to-U)_). Although the method requires a reliable structural model for the folded conformation, which sometimes may not be available, advances in high resolution AI-based protein modeling^44,45^ will likely allow the application of the method to the entire proteome.

## 2 Methods

### 2.1 General MD Simulation Workflow for Calculation of Unfolding Energetics (Δ*H*_unf_, ΔCp_unf_, and Δ*G*_unf_) in Apoproteins

**Figure 1:**
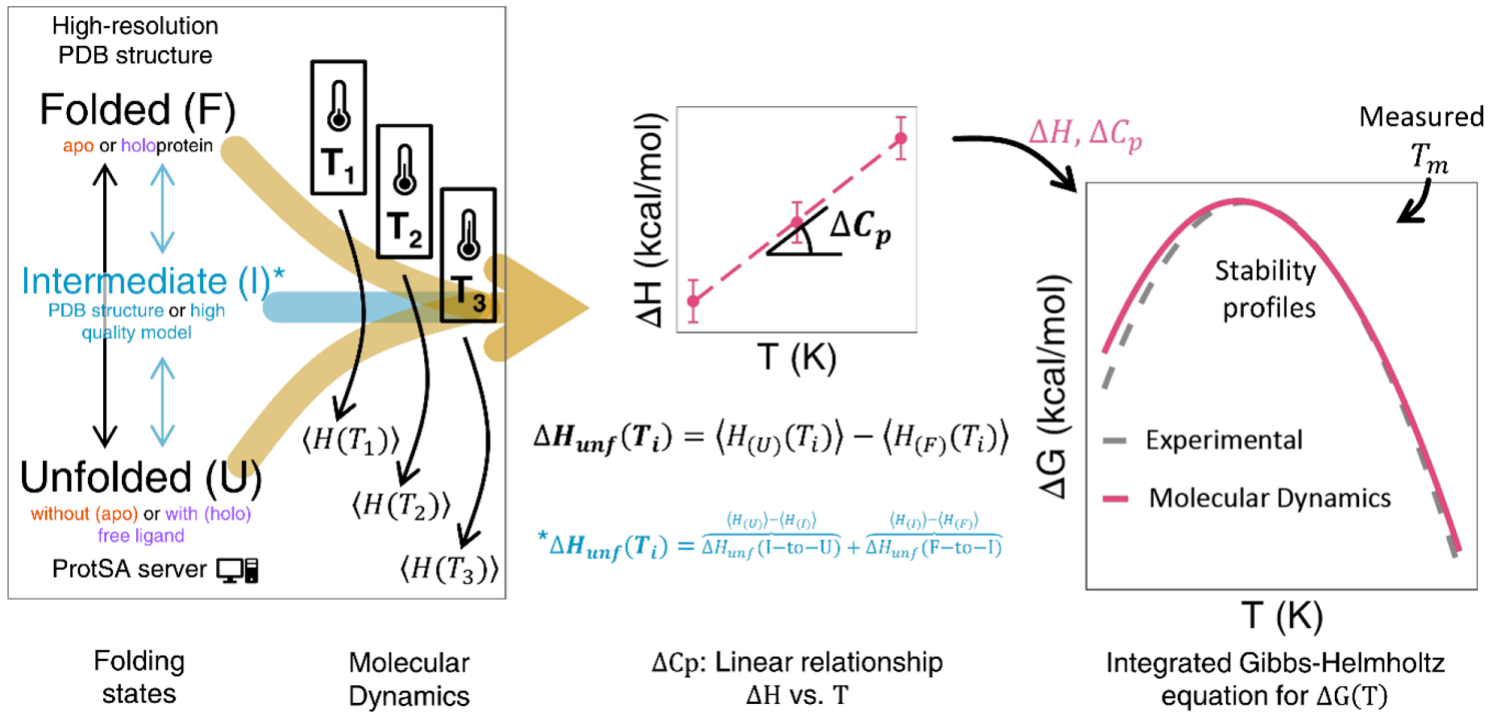
General workflow of the devised MD-based approach. The enthalpy of simulation boxes containing either folded (e.g., *H*_apo(F)_ or *H*_holo(F)_) or unfolded (e.g., H_apo(U)_ or H_apo(U)+cofactor_ in holoproteins) protein or, when applicable, a structure representative of an intermediate state (e.g., *H*_apo(I)_) is directly computed and averaged from MD simulations. The unfolding enthalpy change (Δ*H*_unf_) of interest is obtained as the difference between the enthalpies of the appropriate simulation boxes. The simulations are performed at three temperatures, and the change in heat capacity (ΔCp_unf_) is obtained as the slope of a linear plot of enthalpy change versus temperature. The two calculated thermodynamic changes (Δ*H*_unf_ and ΔCp_unf_) are combined with the experimental *T*_m_ of the protein to calculate the conformational stability by using the Gibbs–Helmholtz equation (eq 1). For holoproteins, a similar equation, SI eq 5 in the Supporting Information, is used that applies a correction to Gibbs free-energy to account for the ligand concentration and uses the van’t Hoff approximation to describe the temperature dependence of the binding constant, *K*_b_(*T*). The number of water molecules and ions present in the folded and unfolded (or intermediate, if applicable) boxes must be identical. Forty replicas of the folded box (normally built from a high-resolution PDB structure) and 100 replicas of the unfolded one (built from a filtered sample of completely unfolded conformations generated by the ProtSA server^25^) are simulated. For intermediate states, 100 simulation replicas were built from a representative structural ensemble. For holoproteins, the unfolded box is built by placing an unfolded protein molecule generated with ProtSA and one molecule of the cofactor at a given minimum distance of the protein. The rest of the general details can be found in Methods and in panel a of Figures 2–4 and Figures S1–S4.

A previous version of the workflow here described has been reported.^24^ The current version (Figure 1) relies on a higher sampling of the folded and unfolded states. Briefly, X-ray crystal structures with the highest resolution and sequence coverage have been retrieved from the RCSB Protein Data Bank (https://www.rcsb.org/,^46,47^ see PDB codes below) and taken as the starting structures for modeling the native (folded) state. When needed, the initial crystal structure has been used to model the amino acid replacement leading to the mutant simulated (e.g., the CI2 Ile76Ala and lysozyme Ile3Glu variants).

**Figure 2:**
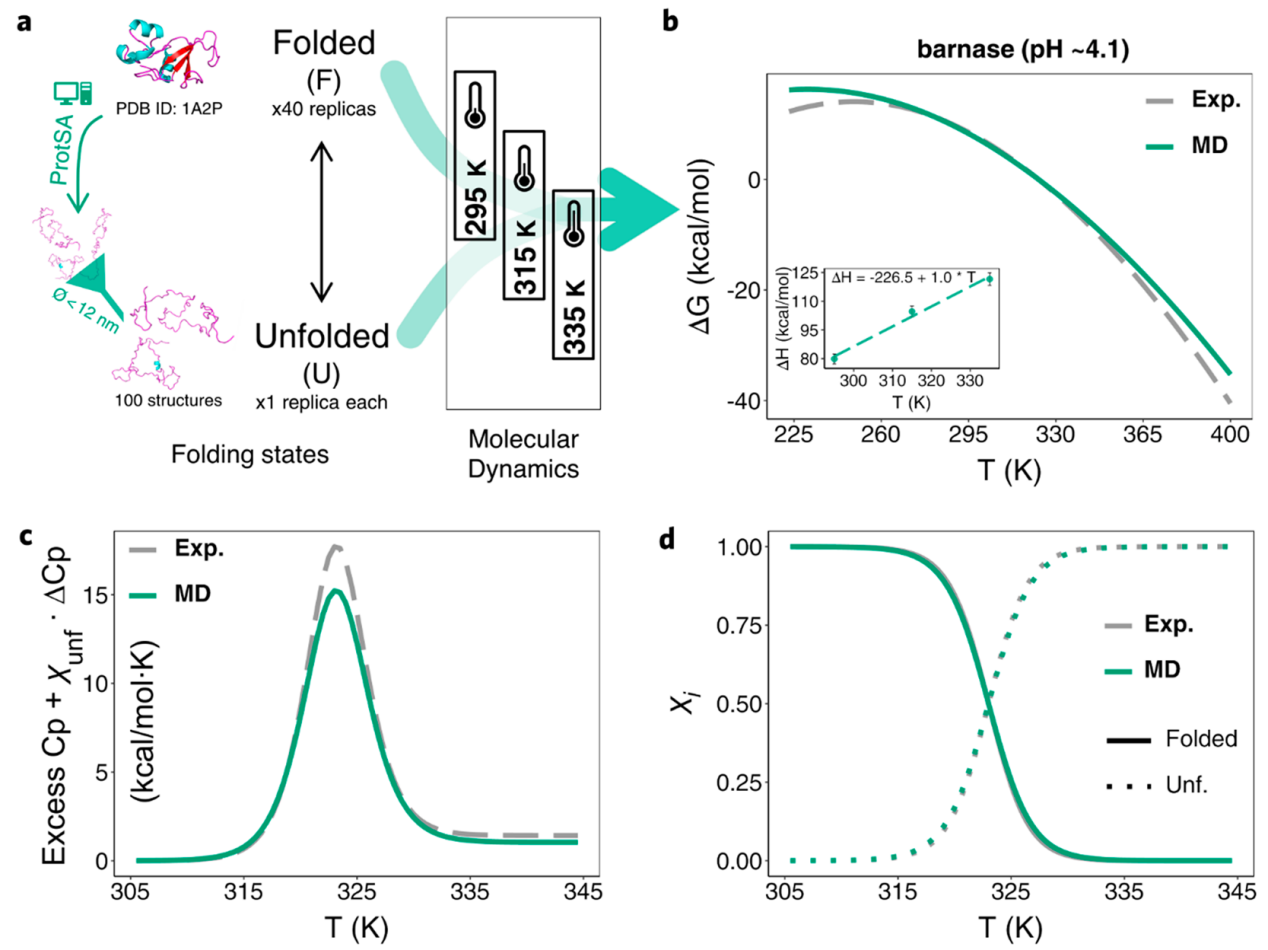
Simplified MD-based scheme and comparison with experimental results for a two-state protein example: barnase. a) The protein models, the number of structures (unfolded) and replicas (folded) simulated, the diameter cutoff used to filter too-elongated unfolded structures obtained from ProtSA^25^ (left, see also Figure S5), and temperatures selected for the MD-based calculation (Charmm22-CMAP) of thermodynamics of barnase. b-d) Stability curves (Δ*G*_unf_(*T*)), thermograms (Excess Cp + χ_unf_ × ΔCp vs *T*), and protein molar fractions (χ_i_) vs *T* plot (*in silico* vs experimental), respectively, obtained for barnase simulated at pH ∼ 4.1. Inset in b depicts the calculated Δ*H*_unf_ vs *T* linear plot with the fitted equation (the slope being ΔCp_unf_) obtained from the MD simulations. The color coding is indicated in the legends of the panels.

**Figure 3:**
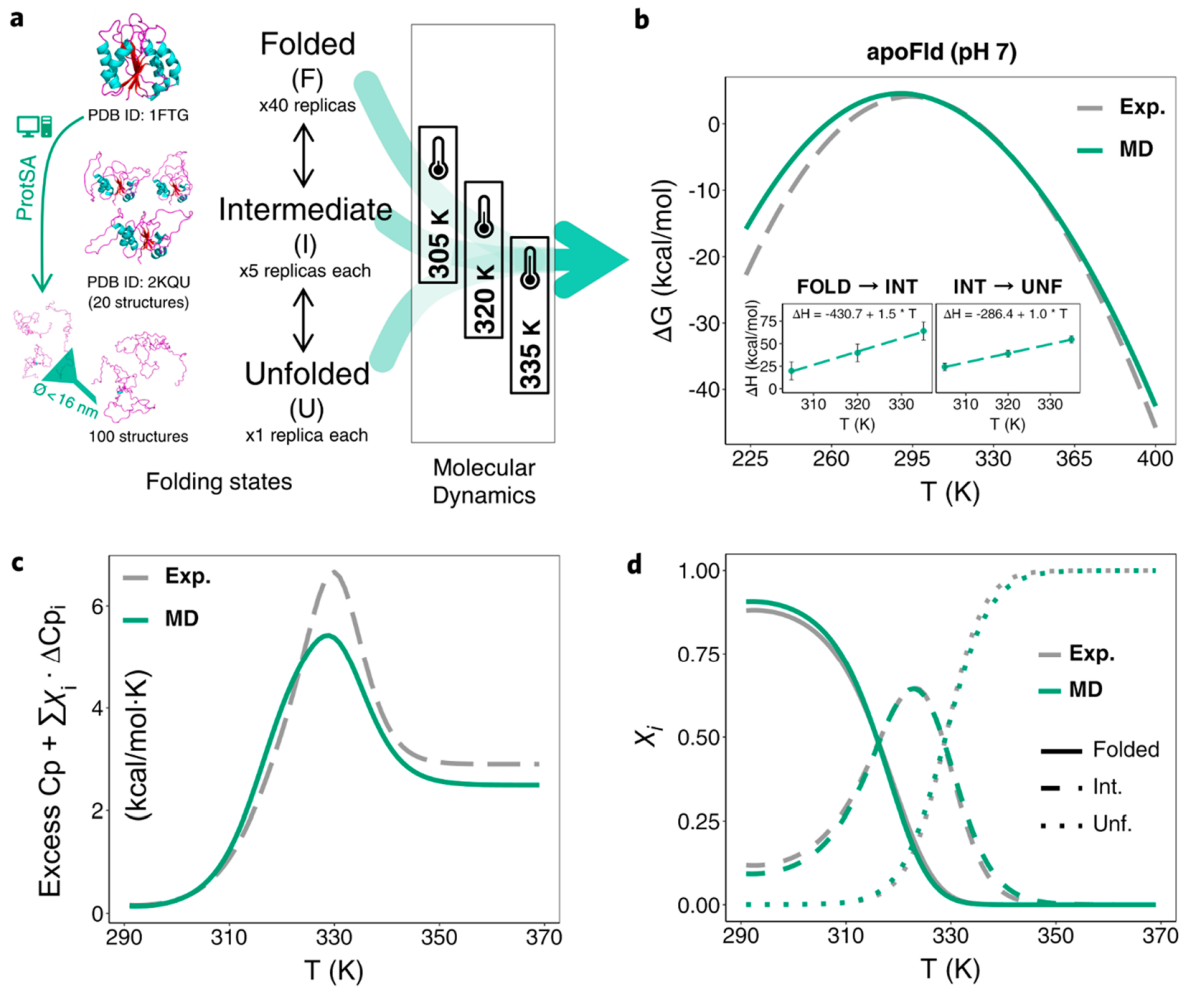
Simplified MD-based scheme and comparison with experimental results for a three-state protein example: apoFld. a) Protein models, number of structures (unfolded) and replicas (folded) simulated, diameter cutoff used to filter too-elongated unfolded structures obtained from ProtSA^25^ (left, see also Figure S5), and temperatures selected for the MD-based calculation (Charmm22-CMAP) of apoFld thermodynamics. b-d) Global stability curves (Δ*G*_unf_(*T*) = Δ*G*_unf(F-to-I)_(*T*) + Δ*G*_unf(I-to-U)_(*T*)), thermograms (Excess Cp + ∑ χ_i_ × ΔCp_i_ vs *T*), and protein molar fractions (χ_i_) vs *T* plot (*in silico* vs experimental), respectively. Inset in b depicts linear plots of calculated Δ*H*_unf_ from the MD simulations vs *T*, with the fitted equation (the slope being ΔCp_unf_) obtained. The color coding is indicated in the legends of the panels.

**Figure 4:**
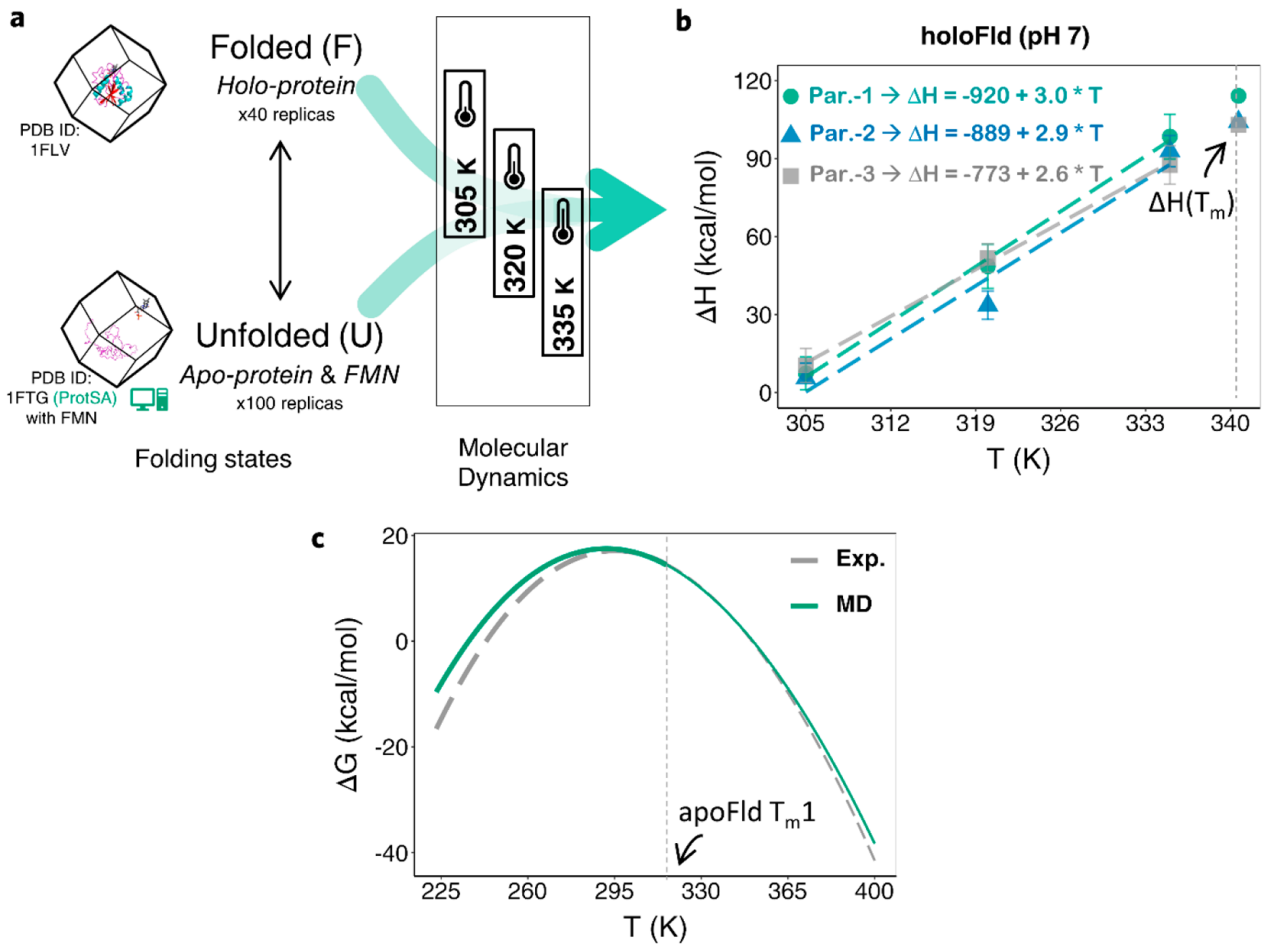
Simplified MD-based scheme and comparison with experimental results for a holoprotein example: holoFld. a) Protein and cofactor models placed in the simulation boxes, folding states, number of structures (unfolded) and replicas (folded) simulated, and temperatures selected for the MD-based calculation (Charmm22-CMAP) of holoFld thermodynamics. b) Calculated Δ*H*_unf_ vs *T* linear plots, with the fitted equations (slopes are the respective ΔCp_unf_) obtained for the three FMN parametrizations tested. Extrapolated Δ*H*_unf_ values at *T*_m_ (340.7 K) are indicated over the vertical dashed line at this temperature. c) Stability curves (Δ*G*_unf_(*T*)) (*in silico* vs experimental) obtained from SI eq 5. Curves appear depicted with finer lines beyond the first *T*_m_ of the apoprotein (316.2 K, Table 1, vertical dashed line) to indicate that in this region the Δ*G*_unf_ values calculated are not reliable. This is so because the van’t Hoff approximation to model the temperature dependence of the binding constant^53^ should work fine as long as the conformation of the protein binding site does not change significantly. However, this will not be the case at temperatures where the apoprotein begins to unfold, and we consider the stability curve of the holoprotein (panel c) to be not reliable beyond the first melting temperature (*T*_m_1) of the apoprotein (316.2 K in the case of apoFld). The fact that at 298.15 K the calculated stability of HoloFld (17.3 ± 2.6 kcal/mol) agrees within error with the stability measured from experimental thermal unfolding curves (19.0 ± 0.9 kcal/mol)^54^ seems to validate the accuracy of the profiles in the range of temperatures below the apoprotein *T*_m_1. Similar to the case of apoproteins, the Δ*H*_unf_ and ΔCp_unf_ values calculated for the holoprotein can be combined with the experimental *T*_m_ to obtain the protein stability curves (Δ*G*_unf_ as a function of temperature). However, as the conformational stability of holoproteins is cofactor concentration dependent, a modified Gibbs–Helmholtz equation that takes into account the binding energetics (SI eq 5, see details in SI Methods) has been derived to calculate the conformational stability as a function of temperature and concentration of free cofactor.

Forty replicas of the folded structure have been simulated, each consisting of a single protein molecule solvated with water molecules in a specified simulation box additionally containing, when required, ions (Na^+^ and/or Cl^–^). On the other hand, a random sample of 100 unfolded structures has been extracted from a large unfolded ensemble (∼2000 structures) generated by the ProtSA server^25^ from the protein sequence (see Figure 1 and panel a in Figures 2–4 and Figures S1–S4). ProtSA uses the Flexible-Meccano algorithm^48^ to generate the backbone-conformation and Sccomp^49^ to add the side chains. Flexible-Meccano uses a coil-library and a simple volume exclusion term to perform conformational sampling, so that the protein unfolded ensembles generated successfully describe backbone fluctuations typically observed in intrinsically disordered proteins (probed by NMR and SAXS experiments).^25^

To avoid using too large simulation boxes, which would increase the simulation time as well as add noise to the results, the most extended unfolded conformations (∼10%) generated by ProtSA have been previously identified and removed as described^24^ (using a diameter-based filtering, Figure S5). The selected 100 unfolded conformations have been simulated in boxes containing one unfolded molecule and exactly the same number of water molecules, ions, and cofactors–when it is the case– as in the corresponding boxes used to simulate the folded conformations of the same protein. For three-state proteins, in addition to the overall enthalpy change, those of the individual steps (F-to-I and I-to-U) can be obtained if the absolute enthalpy of an additional box containing one molecule of protein in the intermediate conformation and the same number of water and ion entities is calculated (see Figure 1 and Figure 3a). To model the intermediate conformation, a suitable structural model is needed. In three-state apoFld, a 20-model NMR ensemble previously described^50^ has been used. In this case, five replicas have been simulated for each of the 20 structures, totaling 100 replicas, the same number of unfolded conformations modeled (Figure 3a).

For each replica, a short 2 ns productive trajectory (see Table S1) has been run, and the individual time-averaged enthalpy (*H*^i^_F_, *H*^i^_U_, or *H*^i^_I_) has been retrieved. The individual enthalpies of replicas of the same conformational state (i.e., folded, unfolded or intermediate) have been ensemble-averaged to obtain the enthalpy corresponding to each folding state (⟨*H*_F_⟩, ⟨*H*_U_⟩, or ⟨H_I_⟩). Subsequently, the unfolding enthalpy change, Δ*H*_unf_, has been calculated by difference, i.e. by subtracting the calculated ensemble-averaged enthalpy obtained from simulations of the folded state from the ensemble-averaged enthalpy obtained from simulations of the unfolded state: Δ*H*_unf_ = ⟨*H*_U_⟩ – ⟨*H*_F_⟩. For three-state proteins, enthalpy changes corresponding to the first unfolding transition (F-to-I) and the second one (I-to-U) have been calculated likewise: Δ*H*_unf(F-to-I)_ = ⟨*H*_I_⟩ – ⟨*H*_F_⟩ and Δ*H*_unf(I-to-U)_ = ⟨*H*_U_⟩ – ⟨*H*_I_⟩ (Figure 1).

The use of multiple short 2 ns simulations in this study is motivated by the well-known overcompaction problem associated with Charmm22-CMAP when long simulations are performed.^24^ We believe that although the sampling of conformational space achieved in an individual 2 ns simulation is limited, the overall sampling obtained by simulating a large and diverse set of starting unfolded structures, as done here (see Section 2.6 below), is adequate.

The calculation of the heat capacity change upon unfolding (ΔCp_unf_) relies on the linear dependency of Δ*H*_unf_ with temperature. For each protein, three not-distant temperatures spanning 30–40 degrees have been selected so that the temperature range covered contains the experimental *T*_m_ of the simulated protein. The three calculated Δ*H*_unf_ values have been represented as a function of simulation temperature, and the ΔCp_unf_ has been calculated as the slope of a linear fit. For three-state proteins (e.g., apoFld), ΔCp_unf(F-to-I)_ and ΔCp_unf(I-to-U)_ have been obtained as the temperature dependence of the calculated enthalpy changes of the corresponding unfolding transition, assuming a linear dependency of Δ*H*_unf_ with temperature (i.e., a temperature independent ΔCp_unf_) is a good and common approximation for performing short extrapolations. However, ΔCp_unf_ is temperature dependent.^51,52^ To assess whether assuming a constant ΔCp_unf_ affects Δ*H*_unf_ extrapolation to *T*_m_, we have additionally calculated barnase Δ*H*_unf_ at six temperatures spanning 100 °C and compared the calculated ΔCp_unf_ and Δ*H*_unf_ extrapolated to *T*_m_ with those obtained as indicated above.

The calculation of the protein stability curves (Δ*G*_unf_ as a function of temperature) has been done through the Gibbs–Helmholtz equation^26^ (eq 1)
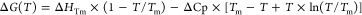
1introducing the calculated Δ*H*_unf_ and ΔCp_unf_ values and the reported experimental *T*_m_.

### 2.2 Specific MD Simulation Workflow for Calculation of Unfolding Energetics (Δ*H*_unf_, ΔCp_unf_, and Δ*G*_unf_) in Holoproteins

In the case of holoproteins (noncovalent complexes of apoprotein and cofactor; e.g. holoFld), the ensemble-averaged enthalpy of the folded (bound) state, ⟨*H*_holo(F)_⟩, has been obtained from simulations (40 replicas) each consisting of one molecule of holoFld solvated with water molecules and ions, as needed (Figure 1 and Figure 4a). Similarly, the energetics of the unfolded (unbound) state has been modeled from simulations (100 replicates) in which one unfolded protein molecule generated with ProtSA^25^ and one cofactor molecule (placed at a minimum distance of 3 nm from the protein) have been put together in a box, where they have been solvated in the same way (Figure 4a). The ensemble-averaged enthalpy of such boxes, ⟨*H*_apo(U)+cofactor_⟩, has been obtained following the averaging scheme of the general workflow. Then, the unfolding enthalpy change has been calculated as Δ*H*_unf_ = ⟨*H*_apo(U)+cofactor_⟩ – ⟨*H*_holo(F)_⟩. As required for this enthalpy change calculation by difference, the number of water molecules and ions in the box containing unfolded protein and cofactor must equal those in the box containing folded holoprotein (Table S2). The simulations have also been performed at three different temperatures, and the unfolding ΔCp_unf_ has been obtained as the slope of a Δ*H*_unf_ versus temperature plot (Figure 4a-b).

### 2.3 Target Proteins and Case Studies

#### 2.3.1 Barnase from *B. amyloliquefaciens* and Nuclease from *S. aureus*

110-Residue barnase^55−59^ and 149-residue nuclease^60−63^ (C-terminal fragment) are well characterized proteins with a two-state equilibrium, as summarized in previous work.^24^ Here, the two-state unfolding energetics of WT barnase and nuclease was determined using the present computational approach. In addition, the reported effect of pH on nuclease stability has been addressed (see Table 1).

**Table 1:**
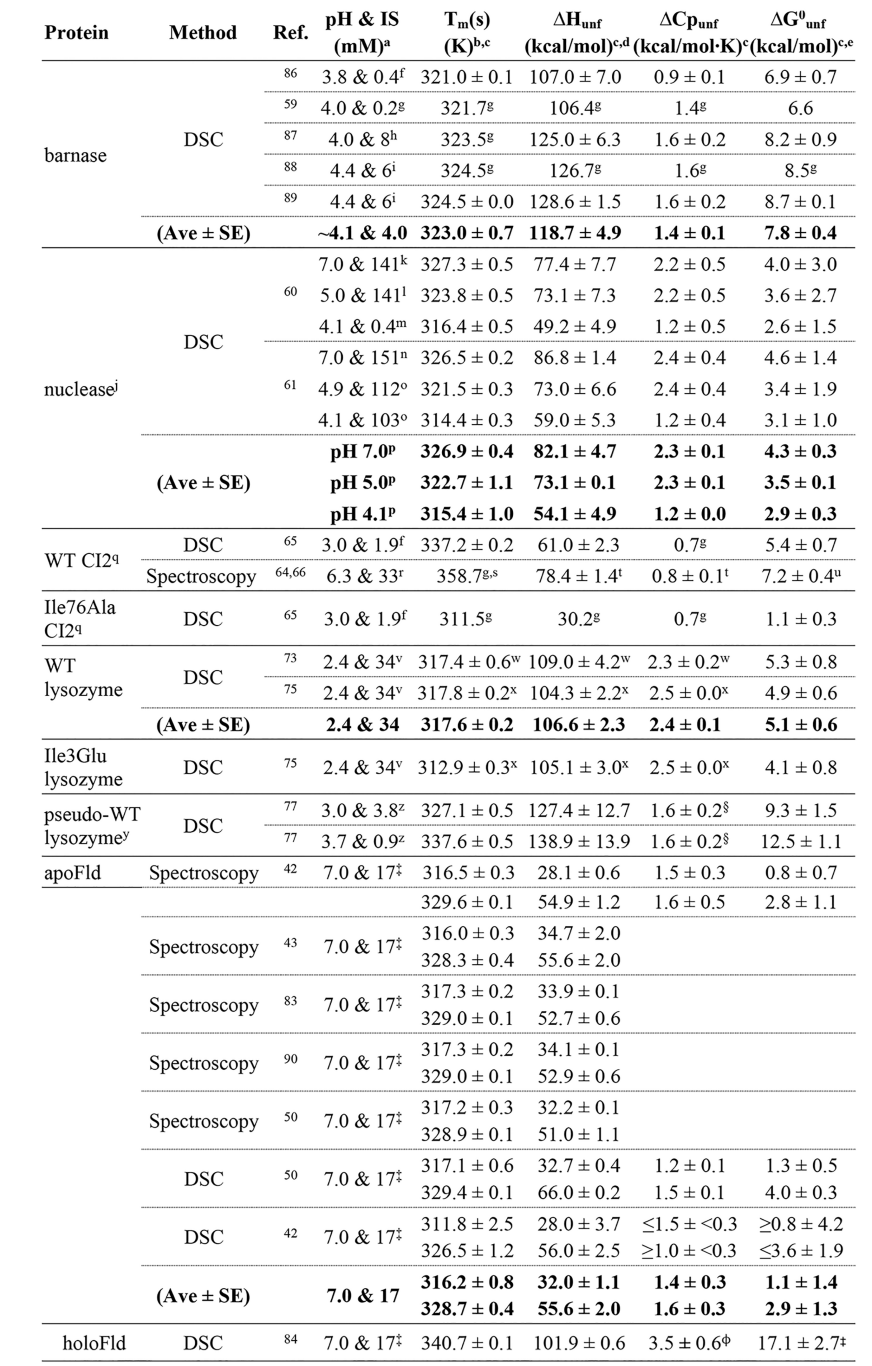
Experimental Thermal Unfolding Data

a Experimental pH and ionic strength (IS) conditions. IS reported or calculated according to buffer, concentration, and pH reported.

b Mid-denaturation temperature (*T*_m_) reported or calculated from a reported empirical equation.

c For three-state apoFld, two values are shown. The first one corresponds to the Native-to-Intermediate transition, and the second one corresponds to the Intermediate-to-Unfolded transition.

d Enthalpy change upon thermal unfolding (Δ*H*_unf_) either reported or calculated from a given empirical equation at *T*_m_.

e Standard (298.15 K) conformational stability (Δ*G*^0^_unf_) obtained from the Gibbs–Helmholtz equation^26^ (eq 1), except otherwise noted. When more than one experimental data are reported, the Δ*G*^0^_unf_ values shown in the “Ave ± SE” row are the average among those values (Ave), and the standard error is obtained by dividing the standard deviation (SD) between the square root of the number of data (SD/√n) (it is not the value calculated through the Gibbs–Helmholtz equation and its propagated associate error). For nuclease, values are calculated at 293.15 K, as experimental data appear reported at that temperature.

f 10 mM glycine hydrochloride. IS calculated from the Henderson-Haselbach equation and the Glycine p*K*_a_ values of 2.37 and 9.78.^91^

g No error reported.

h 50 mM sodium acetate.

i 20 mM sodium acetate.

j The modeled nuclease is the 149-residue C-ter fragment of the protein.

k 20 mM sodium phosphate, 100 mM NaCl, 1 mM EDTA.

l 20 mM sodium acetate, 100 mM NaCl, 1 mM EDTA.

m 20 mM glycine hydrochloride. The influence of salt concentration (between 0 and 800 mM) on measurements seems negligible (see Figure 1c of the reference paper).^60^

n 25 mM sodium phosphate, 100 mM NaCl.

o 20 mM sodium acetate, 100 mM NaCl.

p As measurements of nuclease unfolding thermodynamics are independent of IS^60^ and this parameter largely varied in the experiments reported, the buffer IS is not taken into account in the modeling of this protein.

q Truncated wild-type CI2 and Ile76Ala variant lacking the first 19 amino acid residues.

r 50 mM MES, as reported by Jackson et al.^64^

s *T*_m_ reported by Tan et al.^66^

t Obtained by extrapolating at *T*_m_ after doing a Δ*H*_unf_ vs. *T*_m_ fitting with reported data,^64^ the slope being ΔCp_unf_.

u Value extrapolated to [GdnHCl] = 0 M from thermal denaturation data.^64^

v 20 mM potassium phosphate, 25 mM KCl, 0.5 mM dithiothreitol.

w Values obtained from the reported empirical equations *T*_m_ = 9.63 + 14.41 × pH and Δ*H*_unf_ = 5.97 + 2.33 × *T*. ΔCp_unf_ is the slope of this fitting equation.

x Values obtained from the reported empirical equations *T*_m_ = 9.13 + 14.81 × pH and Δ*H*_unf_ = −10.51 (±0.83) + 2.57 (±0.02) × *T* for the wild-type protein, *T*_m_ = −0.62 (±0.13) + 16.84 (±0.05) × pH and Δ*H*_unf_ = 5.22 (±1.14) + 2.51 (±0.03) × *T* (*T* in Celsius degrees) for the Ile3Glu variant. ΔCp_unf_ is the slope of the Δ*H*_unf_ vs. *T* fitting line.

y Lysozyme variant where residues 54 and 97 appear replaced by a threonine and an alanine, respectively.

z 20 mM glycine hydrochloride. IS calculated from the Henderson-Haselbach equation and the glycine p*K*_a_ values of 2.37 and 9.78.^91^

§ Value obtained from the Δ*H*_unf_ vs. *T* linear fitting plot in Figure 6a of the reference paper.^77^

‡ 50 mM MOPS at 298.15 K.

⧧ Standard Gibbs free-energy of unfolding ([FMN] = 1 M) obtained from SI eq 5 (includes the correction of temperature and ligand concentration, see the SI Methods). For the calculation of this stability, the average (*K*_b_ = 3.61(±1.4) × 10^9^ M) of binding constants reported for FMN,^54,80,84^ as well as the enthalpy (Δ*H*_bind_ = −11.0 ± 0.2 kcal/mol) and heat capacity changes (ΔCp_bind_ = −0.6 ± 0.02 kcal/mol·K) upon binding,^80^ was used. As additional data, a standard Gibbs free-energy change of 19.0 ± 0.9 kcal/mol has been reported by Campos and co-workers.^54^

⌽ ΔCp_unf_ value estimated as follows: sum of ΔCp_unf_ of the two partial unfolding steps of apoFld (1.4 ± 0.3 and 1.6 ± 0.3 kcal/mol·K) plus the ΔCp of binding reported for FMN (−0.6 ± 0.02 kcal/mol·K).^80^

#### 2.3.2 CI2 from Barley Seeds

CI2^37,38^ is a small, 84-residue, globular serine proteinase inhibitory protein extensively studied and reported to fold in a two-state manner as well as to display a two-state thermal unfolding equilibrium.^64−66^ Its 19-residue N-terminal tail is completely unstructured.^67,68^ We have focused here on a truncated form of CI2 lacking the unstructured N-terminal tail because the structure of the full-length protein is not available and because it has been shown that the tail does not contribute to the protein stability.^64,65^ The truncated WT CI2 variant has been modeled at a solvating condition equivalent to pH 3.0 under which experimental energetics is available.^65^ Due to the significantly different thermodynamics quantities reported for WT CI2 at pH 6.3^64,66^ compared to those at pH 3.0 (see Table 1), we have also modeled WT CI2 at pH 6.3 in order to evaluate the sensibility of the method to solvent effects. On the other hand, the CI2 variant Ile76Ala which, relative to WT in identical solvent, shows a significantly lower unfolding enthalpy change and a large destabilization^65^ (Table 1), has been selected to evaluate the feasibility of the approach to calculate the effect of single amino acid replacements on protein stability.

#### 2.3.3 Phage T4 Lysozyme

T4 endolysin (lysozyme)^39^ is a two-domain, 164-residue globular protein that has also been the subject of extensive study and widely used to investigate the role of hydrophobic interactions in protein structural stabilization.^69−71^ Over 500 X-ray structures of T4 lysozyme have been obtained under a variety of experimental conditions (buffer, pH, ionic strength), including those of an engineered pseudolysozyme (see below) and many variants thereof.^46^ WT lysozyme carries two cysteine residues at positions 54 and 97. To ease experimental work on the protein, a Cys54Thr/Cys97Ala variant (termed pseudo-WT lysozyme) has often been studied. WT and pseudo-WT lysozymes^72^ slightly differ in structure and thermodynamics^73−77^ (Table 1). For the sake of testing the method, the energetics of these two lysozyme variants has been calculated. Besides, the energetics of the nonpseudolysozyme variant, Ile3Glu,^75^ has been addressed as a further attempt to capture the effect of single amino acid replacements, and the pseudo-WT lysozyme^77^ has been simulated in different solvent conditions (different pH values) to assess, as with nuclease and CI2, whether the method can capture pH-related effects on protein stability (Table 1).

#### 2.3.4 Anabaena PCC 7119 Flavodoxin (Fld)

Fld^40,78^ is a 169-residue protein that carries electrons from photosystem I to ferredoxin-NADP+ reductase.^79^ Fld capability to transfer electrons is conferred by the presence of a molecule of noncovalently bound FMN cofactor. Reversible removal of the cofactor from the holoprotein (holoFld) leads to the apo form (apoFld). Fld has been widely studied to investigate protein/cofactor interactions,^80,81^ as well as non-native protein conformations.^42,50,82−84^ While apoFld thermal unfolding equilibrium is three-state,^41−43^ binding of FMN greatly stabilizes the complex so that holoFld unfolds following a two-state mechanism.^54,84^ A detailed picture of Fld folding and binding thermodynamics is available.^41−43,50,54,80,83−85^ The reasonably high enthalpy and heat capacity changes (Table 1) of the two apoFld unfolding transitions, namely folded-to-intermediate (F-to-I) and intermediate-to-unfolded (I-to-U), together with the availability of a representative structure of the intermediate conformation^50^ have made us select this protein to test the simulation approach on the calculation of unfolding energetics in three-state proteins.

##### Structure Models (PDB Files) and Coverage

The starting structures used to simulate the folded state of the proteins analyzed have been those with the highest resolution available in the RCSB Protein Data Bank^46,47^ at the time of writing this manuscript, namely the following: 1A2P (1.5 Å resolution)^58^ for barnase, 2SNS (1.5 Å)^92^ for nuclease (C-ter fragment), 2CI2 (2.0 Å)^93^ for CI2 (truncated form), 6LZM (1.8 Å)^72^ for lysozyme, 1L63 (1.75 Å)^94^ for pseudolysozyme, 1FTG (2.0 Å)^95^ for apoFld, and 1FLV (2.0 Å)^96^ for holoFld. On the other hand, the thermal unfolding intermediate state of apoFld has been represented by 2KQU,^50^ a 20-model NMR ensemble of the Phe99Asn mutant previously shown to constitute a reliable representation of this state.^50,82,97^ According to the reference sequences in UniProt,^98^ the structural coverage of the solved sequences is 3-110 (barnase), 83-231 (nuclease C-terminal fragment), 20-84 (WT CI2 and Ile76Ala mutant), 1-162 (WT lysozyme and Ile3Glu mutant), 1-162 (pseudo-WT lysozyme), and 3-170 (apo and holoFld).

### 2.4 Solvation Conditions and MD Simulation General Details

Solvation conditions on the simulated proteins (i.e., protonation states and the number of ions added) have been selected in each case to reproduce the experimental pH and ionic strength (IS) under which the experimental thermodynamics measurements were performed (see detailed information in SI Methods and Table S2). Box dimensions have been adopted from the diameter of the most elongated structure in the unfolded ensemble sampled for a given protein, plus a minimum distance of 1 nm from protein atoms to the simulation box edges. The MD simulation setup has been similar to that previously described^24^ (details are also given in Table S1). All the systems have been simulated with the force field Charmm22 with CMAP correction (version 2.0)^15^ and the explicit water model Tip3p:^27^ the most accurate force field/water model combination reported in previous work.^24^ The Amber99SB-ILDN^16^ force field has been tested again, combined with Tip3p, by modeling the apoFld unfolding thermodynamics. MD simulations have been run and analyzed with Gromacs 2020.^99^ Setting short 2 ns productive trajectories in the workflow^24^ circumvents the known issue of structure overcompaction in long simulations^14,24^ for force fields like Charmm22-CMAP^15^ and Amber99SB-ILDN.^16^ In addition, the simulations performed have been tested for protein overcompaction through the analysis of the evolution of the radius of gyration (Rg) along the trajectories (Table S3). Results of this analysis have confirmed that no significant protein compaction occurs over the trajectories of the systems simulated (Table S3). The mutant variants tested (of CI2 and lysozyme) have been modeled by replacing the wild-type residue by the new one, using the mutator tool of Chimera (v.1.15),^100^ as no solved structures were available. No clashes have been observed in the final mutant structures of the lowest energy obtained after accommodating the new residues, which have been taken as the starting structures in simulations of their folded states. In the case of the apoFld intermediate state, the representative model used (see below) has been mutated back to the wild-type sequence (Chimera v.1.15)^100^ in order to keep the same amino acid sequence as that of the other structural models used in simulations of apo and holoFld. No clashes have been observed after this replacement either. Crystal waters and any other nonprotein molecule have been removed from the PDB structural models chosen (see below).

### 2.5 FMN Parametrization

Three different parametrizations of the FMN molecule (charge −2) have been tested. Namely, ‘Par.-1’ has been obtained ad hoc, assisted by the AmberTools20 package^101^ and the Gaussian 09 program;^102^ ‘Par.-2’ is that reported by Schulten et al.;^103^ and ‘Par.-3’ has been obtained through the SwissParam server.^104^ FMN coordinates have been extracted from the crystal structure of holoFld (PDB ID: 1FLV(96)). For ad-hoc ‘Par.-1’, partial atomic charges have been modeled with Gaussian 09 (HF/6-31G*) and then fitted through the RESP method^105,106^ (with Antechamber),^101,107^ and finally, parameters have been obtained from the General Amber Force Field (GAFF,^108^ Antechamber^101,107^). FMN coordinates have been uploaded to SwissParam^104^ (‘Par.-3’) in mol2 format after adding hydrogen atoms. Except for van der Waals parameters, which have been taken from the closest atom type in Charmm2, parameters and charges with this server derive from the Merck Molecular Force Field (MMFF).^104^

### 2.6 Increased Sampling for Higher Precision

Individual enthalpies (*H*^i^_F_, *H*^i^_U_, or *H*^i^_I_) of the simulated systems (i.e., boxes containing one protein molecule, several ions, and thousands of water molecules) can mount to 10^5^ (negative values) or even higher (see Table S4). These big figures are owed to the large number of water molecules present in the large simulation boxes required to solvate the unfolded conformations. In general, the larger the protein, the larger the negative enthalpy of the simulated box. Therefore, the calculation of unfolding thermodynamics by difference requires a high precision (a low standard error in the calculation) to be able to assess the accuracy of the approach (the difference between experimental and calculated results). Since the enthalpy change of a partial thermal unfolding step of a protein (e.g., the apoFld F-to-I or I-to-U transitions) can be significantly lower than the global enthalpy changes modeled before^24^ (for barnase and nuclease, see Table 1), a higher precision (standard error ≤ 10) than that previously achieved^24^ has been here guaranteed a priori by running a higher number of replicas. For each system (i.e., folded or unfolded), the minimum sample size (40 and 100, respectively) necessary to meet such precision has been estimated as reported.^24^

## 3 Results

### 3.1 Energetics of Two-State Proteins: Barnase, Nuclease, CI2, and Lysozyme

The equilibrium thermal unfolding of barnase, nuclease, CI2, and lysozyme has been described to be two-state. Accordingly, we have calculated their unfolding energetics: Δ*H*_unf_ (at *T*_m_), ΔCp_unf_, and Δ*G*^0^_unf_ (at 25.0 °C or, for nuclease, at 20.0 °C) using the general workflow described in Methods (see Figure 1) where the number of simulated replicas of the folded state and simulated structures in the unfolded ensemble has been increased relative to its initial formulation.^24^ All calculated and experimentally determined Δ*H*_unf_, ΔCp_unf_, and Δ*G*^0^_unf_ values will be reported in kcal/mol, kcal/mol·K, and kcal/mol units, respectively. For simplicity, the units are omitted in this Results section.

Barnase has been simulated (Figure 2a) at pH ∼ 4.1 (Table 1 and Table S2) under solvating conditions similar to those reported in experimental measurements. In previous modeling,^24^ a reasonable agreement was found between experimental and calculated data. Here, the calculated values of Δ*H*_unf_, ΔCp_unf_, and Δ*G*^0^_unf_ obtained with a larger conformational sampling (110.4 ± 3.1, 1.0 ± 0.1, and 7.5 ± 1.2, respectively, Table 2) agree very well with the averaged experimentally determined energetics (118.7 ± 4.9, 1.4 ± 0.1, and 7.8 ± 0.4, Table 1). Due to this fine agreement, the experimental and calculated temperature dependencies of Δ*G*_unf_ (stability curve, Figure 2b), (thermogram, Figure 2c), and state fractions (Figure 2d) nearly coincide. The agreement between experimental and calculated magnitudes is better than that obtained with a smaller sampling (92.3 ± 5.7, 0.9 ± 0.1, and 6.5 ± 0.8, respectively) in the previous calculation.^24^

**Table 2:**
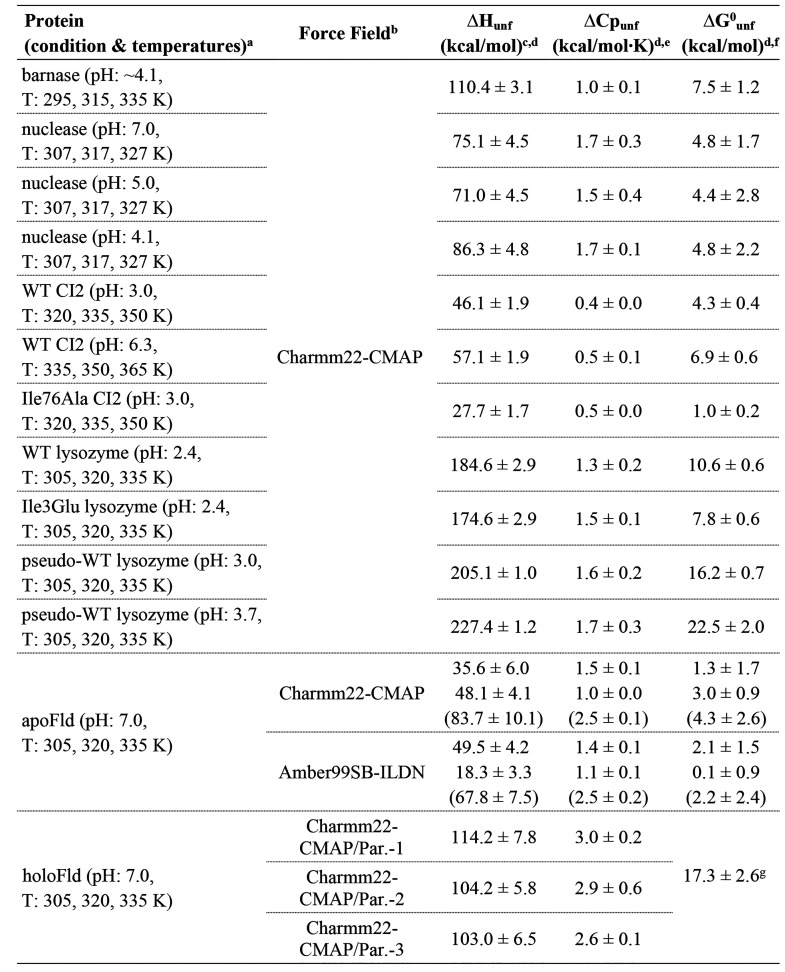
Calculated Thermal Unfolding Energetics from MD Simulations

a Δ*H*_unf_ is calculated at the three indicated temperatures. ΔCp_unf_ obtained as the slope of a Δ*H*_unf_ vs. *T* linear plot.

b Force fields tested for the calculation, and FMN parametrizations used in holoFld systems (see Methods). The water model used is always Tip3p, as described in Methods.

c Calculated enthalpy change upon thermal unfolding (Δ*H*_unf_) at *T*_m_ (see values in Table 1), obtained by extrapolation. Given errors are standard error (SE) obtained as the sum of the SE from folded simulations (40 replicas) plus the SE from unfolded simulations (100 replicas) (see Table S4).

d For three-state apoFld, three calculated Δ*H* values are shown. The upper one corresponds to the enthalpy change of the Native-to-Intermediate transition; the intermediate one corresponds to the enthalpy change of the Intermediate-to-Unfolded transition; and the lower one (between parentheses) corresponds to the total Δ*H*_unf_, obtained by adding up the values calculated for each transition. Likewise, in the ΔCp_unf_ and Δ*G*^0^_unf_ columns, the three values indicated correspond (from top to bottom) to the Native-to-Intermediate, Intermediate-to-Unfolded, and global (Native-to-Unfolded) heat capacity or Gibbs free-energy changes, respectively.

e Calculated ΔCp_unf_ obtained as the slope of a Δ*H*_unf_ vs. *T* linear plot. Fitting errors are given as SE.

f Unfolding Gibbs free-energy changes at 298.15 K calculated using the Gibbs–Helmholtz equation^26^ (or SI eq 5 for HoloFld; see SI Methods). For nuclease, the temperature of reference used, 293.15 K, is the one at which most of the experimental data are reported (Table 1). Given errors are SE obtained by error propagation through the Gibbs–Helmholtz equation^26^ (or SI eq 5 for HoloFld).

g Standard Gibbs free-energy (at 1 M FMN) calculated through SI eq 5 (SI Methods and the footnote *bb* in Table 1).

Alternatively, barnase ΔCp_unf_ has been calculated from a linear fit of not just 3 but 6 Δ*H*_unf_ values newly obtained from MD simulations spanning 100 °C (from 275 to 375 K). The value and error obtained for ΔCp_unf_ are the same (1.0 ± 0.1), and the calculated Δ*H*_unf_ at *T*_m_ is 100.1 ± 2.2, which is close to the value of 110.4 ± 3.1 previously obtained. Considering the two calculations as independent experiments and using only the data obtained in the common temperature interval, the average values and standard errors obtained for ΔCp_unf_ and Δ*H*_unf_ at *T*_m_ are 1.1 ± 0.1 and 106.3 ± 4.0, respectively. The standard errors obtained are only slightly bigger than those reported in Table 2, obtained from a single calculation using Δ*H*_unf_ at three temperatures. On the other hand, we have noticed that the Δ*H*_unf_ versus *T* plot spanning 100 °C shows a slight departure from linearity (Figure S6) as expected if ΔCp_unf_ is not constant.^51,52^ Because the experimental information on the temperature dependence of ΔCp_unf_ is lacking for most of the proteins analyzed here, both the calculated and experimental stability curves displayed in Figures 2–4 and Figures S1–S4 are obtained from eq 1 or SI eq 5 (Figure 4), using constant ΔCp_unf_ values, either experimental or calculated.

Nuclease unfolding thermodynamic data are available over a range of pH (from 3 to 8.5) and solvating conditions.^60,61^ WT nuclease has been simulated (Figure S1a) at three pH values: 7.0, 5.0, and 4.1 (see solvating conditions and protonation states in Table 1 and Table S2). At pH 7.0, the calculated Δ*H*_unf_, ΔCp_unf_, and Δ*G*^0^_unf_ values (75.1 ± 4.5, 1.7 ± 0.3, and 4.8 ± 1.7, respectively, Table 2) match very well the averaged experimental ones (82.1 ± 4.7, 2.3 ± 0.3, and 4.3 ± 0.3, Table 1). This excellent agreement is reflected, as seen for barnase, in a fine correspondence between the experimental and calculated temperature dependences of the Gibbs free-energy difference, thermogram, and molar fractions (Figure S1b-d). The second solvating condition simulated for nuclease reproduces a protonation scheme previously used,^24^ corresponding to pH 5.0. Under this condition, our calculated energetics (Δ*H*_unf_ = 71.0 ± 4.5, ΔCp_unf_ = 1.5 ± 0.4, and Δ*G*^0^_unf_ = 4.4 ± 2.8, Table 2) matches fairly well the experimental values (73.1 ± 0.1, 2.3 ± 0.1, and 3.5 ± 0.1, respectively, Table 1 and Figure S1e-f). The application here of a more exhaustive sampling yields results for nuclease that are as accurate as those obtained for this protein with a smaller sampling in previous work (Δ*H*_unf_ = 76.0 ± 8.1, ΔCp_unf_ = 1.8 ± 0.1, and Δ*G*^0^_unf_ = 4.6 ± 1.4).^24^ Nuclease stability is thus accurately calculated in the pH range 5.0–7.0. At lower pH (pH 4.1), however, the method overestimates Δ*H*_unf_ and ΔCp_unf_, which leads to a less accurate calculated stability (4.8 ± 2.2, Table 2) compared to the experimental value (2.9 ± 0.3, see Table 1 and Figure S1g-h).

Thermodynamic data for chymotrypsin inhibitor 2 (WT truncated form, see Methods) and for a broad set of point mutants analyzed under different solvation conditions (varying in pH and ionic strength) are available^64−66^ (Table 1). Here, WT CI2 has been simulated (Figure S2a) at two pH conditions for which reliable experimental data are reported (Table 1 and Table S2). At pH 3.0, the calculated Δ*H*_unf_ and ΔCp_unf_ values (46.1 ± 1.9 and 0.4 ± 0.03, respectively) are a bit lower than the corresponding experimental values (61.0 ± 2.3 and 0.72). Notwithstanding, the calculated Δ*G*^0^_unf_ at this pH (4.3 ± 0.4) virtually agrees within error of the experimental stability (5.4 ± 0.7). At pH 6.3, CI2 is more stable than at pH 3.0, as the experimental Δ*H*_unf_ and ΔCp_unf_ values (78.4 ± 0.7 and 0.8 ± 0.1, respectively) combine to a higher conformational stability (Δ*G*^0^_unf_ = 7.2 ± 0.4). The higher experimental Δ*H*_unf_ and ΔCp_unf_ values at pH 6.3 relative to pH 3.0 are captured by our simulations (calculated values at pH 6.3:57.1 ± 0.5 and 0.5 ± 0.07), and so is the increase in conformational stability (calculated value at pH 6.3: 6.9 ± 0.6). We have also assessed the capability of the simulation approach to detect changes in stability associated with point mutations. For that, we have computed the energetics of the Ile76Ala CI2 variant at pH 3.0 and compared it to that of WT CI2 at the same pH. Substitution of the bulky WT isoleucine residue by alanine creates a cavity that severely destabilizes the folded structure of the mutant. The reduced stability of Ile76Ala CI2 compared to WT is evidenced in its experimental unfolding energetics (Δ*H*_unf_ = 30.2, ΔCp_unf_ = 0.7, and Δ*G*^0^_unf_ = 1.1 ± 0.3, Table 1), which is accurately obtained from our simulations (27.7 ± 1.7, 0.5 ± 0.01, and 1.0 ± 0.2, respectively, Table 2). Thus, the simulation workflow allows capture of the experimental observations that 1) WT CI2 is stabilized by raising the pH from 3.0 to 6.3 (experimental ΔΔ*G*_unf(pH3→pH6.3)_ = +1.8 ± 1.1; calculated value = +2.5 ± 1.2) and 2) WT CI2 is severely destabilized by replacing Ile76 by Ala (experimental ΔΔ*G*^0^_unf(WT→I76A)_ = −4.3 ± 1.0; calculated value = −3.3 ± 0.6). Experimental and calculated stability curves, thermograms, and state fractions of WT (pH 3.0), WT (pH 6.3), and Ile76Ala CI2 mutant (pH 3.0) are compared in Figure S2b-h. A good agreement between calculated and experimental data can be observed, which is particularly remarkable for the Ile76Ala CI2 variant (Figure S2g-h).

The thermal stability of WT lysozyme and many variants thereof have been reported.^73−76^ Lysozyme has been simulated here (Figure S3a) at pH 2.4 (WT and Ile3Glu mutant) and at pH 3.0 and 3.7 (pseudo-WT; Figure S4a). The experimental ΔCp_unf_ is accurately calculated for the pseudo-WT but underestimated for the WT. For the four simulated lysozyme variants or pH conditions (Table 1), the calculated Δ*H*_unf_ values (Table 2) clearly overestimate the corresponding experimental ones (Table 1). As a consequence, the stabilities calculated also overestimate the experimental values, and the stability temperature dependencies (Figures S3b-f and S4b-f) do not match the calculated ones. Thus, the actual lysozyme stabilities are not correctly calculated. Possible reasons for this are indicated in the Discussion section. Still, both the lower stability of the Ile3Glu mutant relative to WT at pH 2.4 (ΔΔ*G*^0^_unf(WT→Ile3Glu)_ = −1.0 ± 1.4) and the higher stability of pseudo-WT at pH 3.7 compared to pH 3.0 (ΔΔ*G*_unf(pH3.0→pH3.7)_ = +3.2 ± 2.6) are qualitatively captured (−2.8 ± 1.2 and +6.3 ± 2.7, respectively).

### 3.2 Energetics of a Three-State Protein: apoFld

ApoFld thermal unfolding equilibrium is three-state, with a well-defined intermediate accumulating at equilibrium with the folded and unfolded conformations. For this protein, the unfolding enthalpy changes of the sequential partial unfolding equilibria (F-to-I and I-to-U) have been separately calculated using the general workflow (Figure 1). Structures or ensembles (see Methods) representing the three states involved in the transitions have been simulated (Figure 3a). The results show that the calculated enthalpy changes of the two unfolding transitions, Δ*H*_unf(F-to-I)_ = 35.6 ± 6.0 and Δ*H*_unf(I-to-U)_ = 48.1 ± 4.1 (Table 2), are in excellent agreement with the corresponding experimental enthalpies of 32.0 ± 1.1 and 55.6 ± 2.0 (Table 1). The heat capacity changes calculated for each partial unfolding step, ΔCp_unf(F-to-I)_ = 1.5 ± 0.1 and ΔCp_unf(I-to-U)_ = 1.0 ± 0.0, respectively (2.5 ± 0.1 for the global transition, Table 2), are also in fair agreement with the experimental values of 1.35 ± 0.3 and 1.55 ± 0.3, respectively (2.9 ± 0.6 for the global transition, Table 1). From these calculated data and the corresponding experimental *T*_m_s (Table 1), the Gibbs free-energy changes of the individual apoFld unfolding transitions are calculated at 25.0 °C using the Gibbs–Helmholtz equation^26^ (eq 1), and the global apoFld stability is then obtained as the sum of the individual free-energy changes. A fine correspondence between the calculated stability values, Δ*G*^0^_unf(F-to-I)_ = 1.3 ± 1.7, Δ*G*^0^_unf(I-to-U)_ = 3.0 ± 0.9, and Δ*G*^0^_unf(F-to-U)_ = 4.3 ± 2.6 (Table 2), and the corresponding experimental ones, 1.1 ± 1.4, 2.9 ± 1.3, and 4.0 ± 2.7, is observed. The outstanding correspondence between calculated and experimentally determined apoFld thermal unfolding thermodynamics is also observed in the compared stability curves, thermograms, and folded/intermediate/unfolded state fractions depicted in Figure 3b-d.

An otherwise identical calculation of apoFld thermal unfolding thermodynamics has been carried out using the Amber99SB-ILDN force field instead of Charmm22-CMAP. Although accurate heat capacity changes have been calculated with Amber99SB-ILDN for the two equilibria (1.4 ± 0.1 and 1.1 ± 0.1, respectively, Table 2), the calculated enthalpy changes (Table 2) do not agree well with the experimental values (Table 1), which results in less accurate calculations of the individual Gibbs free-energy changes (Table 2) compared to those obtained with Charmm22-CMAP. For barnase and nuclease, the better agreement of Charmm22-CMAP thermodynamics calculations with experimental values compared to calculations with Amber99SB-ILDN was already reported.^24^

### 3.3 Energetics of a Holoprotein: holoFld

The calculation of the thermal unfolding energetics of a holoprotein (a protein carrying a noncovalently bound cofactor) has been performed as described in Methods and illustrated in Figure 4a. To model holoFld energetics, three different FMN parametrizations have been tested (see Methods). Δ*H*_unf_ calculated for holoFld with any of them (ranging from 103.0 ± 6.5 to 114.2 ± 7.8, Table 2) is in fair agreement with the experimental value reported by Lamazares and co-workers^84^ from DSC measurements (101.9 ± 0.6, Table 1).

holoFld ΔCp_unf_ has not been reported, but an estimation can be done by adding the reported value for FMN dissociation (ΔCp_diss_ = −ΔCp_bind_ = 0.6 ± 0.0)^80^ to the apoFld ΔCp_unf_ (2.9 ± 0.6, Table 1). Thus, the holoFld ΔCp_unf_ is estimated to be 3.5 ± 0.6. Our calculated holoFld ΔCp_unf_ values (reported in Table 2 and depicted as the slope of fitting lines in Figure 4b) indicate that ΔCp_unf_ obtained with either FMN Par.-1 or FMN Par.-2 (3.0 ± 0.2 and 2.9 ± 0.6, respectively) agrees within experimental error, and that obtained with FMN Par.-3 (2.6 ± 0.1) while lower is still above the value previously calculated for apoFld (2.5 ± 0.1, Table 2), in agreement with the observed positive value of ΔCp_diss_.

The stability of holoFld at 25.0 °C is obtained through SI eq 5 (see derivation in SI Methods). To the apoprotein Gibbs free-energy, SI eq 5 applies a correction due to the ligand concentration and incorporates the van’t Hoff approximation^53^ to account for the temperature dependence of the binding constant. Thus, SI eq 5 is not based on the thermodynamics derived from the holoFld simulations but on those of the apoprotein (Δ*H*_apo(unf)_, ΔCp_apo(unf)_) plus the cofactor energetics. Using SI eq 5, the Δ*G*^0^_unf_ value calculated (17.3 ± 2.6, Table 2) is in close agreement with the experimental value (17.1 ± 2.7, Table 1) similarly obtained with SI eq 5 using experimental Δ*H*_apo(unf)_ and ΔCp_apo(unf)_ data. Importantly, the calculated Δ*G*^0^_unf_ also matches, within error, the experimental stability of holoFld directly obtained from thermal unfolding curves (19.0 ± 0.9).^54^

## 4 Discussion

The devised MD simulation workflow allows for the calculation of Δ*H*_unf_, ΔCp_unf_, and Δ*G*_unf_, i.e., three of the main thermodynamic magnitudes governing the stability of proteins. The overall accuracy of the method can be assessed from lineal plots of calculated versus experimentally determined values of each of those magnitudes.

**Figure 5:**
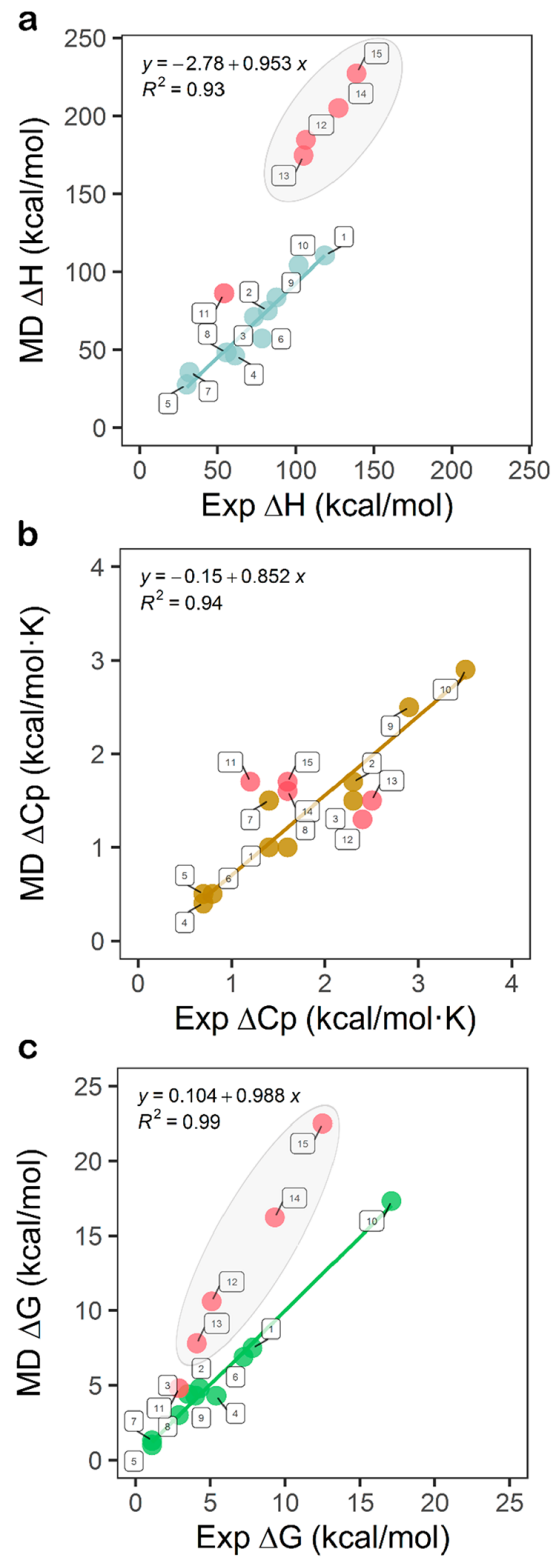
Global assessment of the approach for calculation of unfolding thermodynamics with Charmm22-CMAP/Tip3p. a) Scatter plot of MD-calculated vs experimental Δ*H*_unf_ for the set of proteins simulated (including different solvating conditions and variants). The linear fit shown in this panel (also in panels b and c) was performed over the following ten systems: barnase at pH ∼ 4.1 (dot number 1 in legend), nuclease at pH 7.0 (2) and pH 5.0 (3), WT CI2 at pH 3.0 (4), Ile76Ala CI2 at pH 3.0 (5), WT CI2 at pH 6.3 (6), apoFld(F-to-I) (7), apoFld(I-to-U) (8), apoFld(F-to-U) (9), and holoFld(FMN Par.-2) (10). The fitting equation and the square Pearson correlation coefficient are given. b) Scatter plot and linear fit of MD-calculated vs experimental ΔCp_unf_. c) Scatter plot and linear fitting of MD-calculated vs experimental protein stability (Δ*G*^0^_unf_ at 298.15 K for all proteins except for nuclease that is compared at 293.15 K). Experimental values (*x*-axis) are the averages (or individual value in some cases) of data obtained from the literature, as summarized in Table 1, while calculated values are those presented in Table 2. Red circles represent outliers (or cases treated as such, see the Results and the Discussion sections) not considered in the linear fitting, namely the following: nuclease at pH 4.1 (dot number 11 in legend), WT lysozyme at pH 2.4 (12), Ile3Glu lysozyme at pH 2.4 (13), pseudo-WT lysozyme at pH 3.0 (14), and pseudo-WT lysozyme at pH 3.7 (15). In panels a and c, the 4 outliers of lysozyme and pseudolysozyme systems are enclosed in a semitransparent gray oval to visualize them as similar systems whose enthalpy change upon unfolding (Δ*H*_unf_) and protein stability (Δ*G*_unf_) are all overestimated by our simulations. Out of the three setups tested for holoFld, the results obtained with FMN parametrization 2 (the most accurate one, see Tables 1 and 2) are depicted.

The primary figure calculated is the unfolding enthalpy change (Δ*H*_unf_) of the proteins investigated. With the exception of lysozyme (simulated in four conditions) and nuclease (when simulated at low pH, pH 4.1), which are clear outliers, the linear plot (Figure 5a) can be fitted to a straight line with an ordinate close to zero (−2.8), slope close to unity (0.95), and a correlation of R^2^ = 0.93. The fitting includes the data from ten simulated systems (barnase, nuclease at two pH values, and two partial unfolding equilibria, as well as the whole transition of three-state apoFld, CI2 at two pH values plus one mutant, and holoFld) spanning a range of Δ*H*_unf_ values from 30 to 120 kcal/mol. It is thus clear that Δ*H*_unf_ can be accurately calculated by using this approach.

The second figure is the unfolding heat capacity change (ΔCp_unf_), which is also captured for the 10 protein systems well fitted in Figure 5a. The four lysozyme systems simulated (WT, a variant of WT, and a pseudo-WT variant at two pHs), as well as nuclease at pH 4.1, fit worse than the other 10 systems (Figure 5b). Albeit their calculated ΔCpunf values do not differ too much from their experimental ones, they have been treated as outliers for consistency. The linear fit with data from the other 10 simulated systems yields a straight line with an ordinate close to zero (−0.15), slope close to unity (0.85), and a correlation of R^2^ = 0.94, indicating that the change in heat capacity of unfolding can be also calculated in an accurate manner. The range of ΔCp_unf_ values spanned in the plot goes from 0.6 to 3.5 kcal/mol·K.

The third figure is the unfolding Gibbs free-energy change (Δ*G*_unf_), i.e., the conformational stability of the protein. To derive it, the workflow combines the calculated enthalpy and heat capacity changes with experimental values of melting temperatures, using the Gibbs–Helmholtz equation (eq 1) for apoproteins, or an analogous equation (SI eq 5) for holoproteins. As expected, in the linear plot of calculated versus experimentally determined stabilities (Figure 5c) lysozyme yields outliers, as the high enthalpy changes calculated for this protein system are carried over in the calculation of the stability. Although nuclease at pH 4.1 is not a clear outlier in the stability representation, it has been kept as such for consistency. The fitting of the calculated and experimental values for the other 10 systems simulated gives rise once again to a straight line with close to zero intercept (0.10), close to unity slope (0.99), and a high correlation of R^2^ = 0.99. It seems thus that protein conformational stability can be accurately calculated from first-principles using the described simulation workflow. The range of Gibbs free-energies spanned in the plot goes from 1 to 17 kcal/mol.

The MD simulation workflow accurately calculates the protein changes in enthalpy, heat capacity, and Gibbs free-energy upon unfolding and can also be used to compare the stability of a protein under different pH values or to compare the stability of a wild-type protein with that of its mutants. According to our literature search, no similar approach for the calculation of protein folding energetics has been described, which precludes a direct comparison of our approach with other methods. The systems successfully calculated here contain representatives of the main protein classes (mainly alpha, mainly beta, and alpha beta),^109^ with sequences ranging from 84 to 169 residues, and isoelectric points from 4.0 to 8.9. They include proteins that undergo two- or three-state thermal unfolding as well as proteins that do or do not carry a tightly bound cofactor. Altogether, these proteins offer a fair representation of natively folded proteins, for which the unfolding process leads to fully unfolded conformations. Detailed thermodynamic studies on much larger proteins are scarce, and the approach has not been tested on large proteins. We foresee no reasons why the energetics of larger proteins cannot be calculated with similar accuracy using sufficient sampling, provided that they adopt fully unfolded conformations after heating. Full unfolding of the denatured state is a requisite, as it is necessary to be able to build realistic models of the unfolded ensemble using ProtSA.^25^

For one of the proteins simulated, lysozyme, the calculations have consistently led to overestimated Δ*H*_unf_ values, which has translated to overestimated stability. In principle, the method could have failed for this protein due to insufficient quality of the models used to represent its folded and unfolded conformations. This is unlikely, however, as the folded structures have been solved in a highly experience lab,^110^ and they get good marks (not shown) when subjected to quality control with the MolProbity server.^111^ On the other hand, the model of the unfolded ensemble generated by ProtSA^25^ would be wrong if the lysozyme unfolded state were compact, but we have found no reports pointing to that. A different possible reason for the inaccurate lysozyme calculation may be small inaccuracies in force field parameters. Although the same force field has been used in lysozyme and in the successfully calculated proteins, it should be noticed that force field parameters are globally optimized, and optimal individual performance from each parameter cannot be taken for granted. In this respect, of all the systems simulated here, lysozyme stands out as the one containing the highest net (positive) charge (Table S2), only paralleled by the high net (positive) charge of nuclease under the simulation condition of pH 4.1, where inaccurate results have also been obtained. It is thus possible that the discrepancy between calculated and experimental lysozyme unfolding magnitudes is related to insufficient tuning of Coulombic treatment by the Charmm22-CMAP force field^15^ for lysine and arginine protonated side chains. Alternatively, or in addition to this, some uncertainty in the protonation state of lysozyme carboxyl groups at the acidic pH of the simulations could contribute to inaccuracy. Whatever the reason, the poorer performance of the method on lysozyme suggests that it should be used with caution when highly positively charged proteins are simulated at acidic pH values. As proteins are rarely studied experimentally under basic pH conditions, we have not tested the performance of the method at high pH values.

Although the described approach is based on a specific force field and water model, it suggests that current force fields are already close to capturing the complexity of the protein folding energetics. We hope that our results will encourage further improvement of the force fields and water models. Toward that goal, the described methodology constitutes an effective and efficient way to assess the ability of a given force field to replicate the changes in energy that govern protein equilibria.

## 5 Conclusions

The energetics (folding Δ*H* and ΔCp) of two- and three-state proteins (with or without bound cofactors) can be accurately computed using conventional force fields and water models by sampling the unfolded ensemble energy with many short MD simulations of conformationally diverse starting structures. If the melting temperature of the simulated protein is known, the stability curve providing the value of Δ*G* as a function of the temperature can also be obtained. Besides, smaller stability differences (ΔΔ*G*) due to differences in solution conditions (e.g., differences in pH value) or caused by point mutations can be semiquantitatively obtained. However, the combination of force field and water model used here (which is nevertheless better than other combinations based on force fields specifically tuned to avoid overcompaction) overestimates Δ*H* in the case of highly charged proteins if they are simulated at low pH. We propose that the thermodynamic approach described here for calculating protein energetics from MD simulations can be of help to force field developers to fine-tune force fields and water models, which, until now, have paid great attention to reproducing geometric and dynamical features of proteins but little attention to reproducing the energy changes governing protein equilibria.

## Data Availability

The files used/necessary for the calculations done in this work using Molecular Dynamics simulations can be downloaded from https://zenodo.org/record/8165111.

## Supporting Information Available

Additional methods, MD simulations setup, and experimental details. Data of calculated thermodynamics for additional protein systems simulated compared with their experimental values, including stability curves, thermograms, and protein molar fractions plots (PDF)

## Abbreviations

AI: Artificial Intelligence
DSC: Differential Scanning Calorimetry
FMN: Flavin Mononucleotide
IS: Ionic Strength
LEM: Linear Extrapolation Method
MD: Molecular Dynamics
NMR: Nuclear Magnetic Resonance
SAXS: Small-Angle X-ray Scattering
NPT: Isothermal–isobaric ensemble in MD simulations
NVT: Canonical ensemble in MD simulations
PBC: Periodic Boundary Conditions
PME: Particle Mesh Ewald
Rg: Radius of gyration
SE: Standard Error
