# Supplementary material for: Calculation of Protein Folding Thermodynamics Using Molecular Dynamics Simulations
Source: J Chem Inf Model. 2023 Nov 13;63(24):7791–806. doi: 10.1021/acs.jcim.3c01107 (PMC10751793; doi:10.1021/acs.jcim.3c01107)
Supplement: Supplementary file 1 — ci3c01107_si_001.pdf [file ci3c01107_si_001.pdf]

Supporting Information for

Calculation of Protein Folding Thermodynamics using Molecular Dynamics Simulations

Juan José Galano-Frutos[1, 2], Francho Nerín-Fonz[1, §], Javier Sancho[1, 2, 3]

[1]Department of Biochemistry, Molecular and Cell Biology. Faculty of Science, University of Zaragoza, 50009 Zaragoza, Spain

[2]Biocomputation and Complex Systems Physics Institute (BIFI). Joint Unit GBs-CSIC, University of Zaragoza, 50018 Zaragoza, Spain

[3]Aragon Health Research Institute (IIS Aragón), 50009 Zaragoza, Spain

[§]Current Address: Biomedical Research Foundation, Academy of Athens, Athens 11527, Greece

Corresponding author: Javier Sancho, E-mail: jsancho@unizar.es

Content

SUPPORTING INFORMATION METHODS

Simulated Solvating Conditions.

Calculation of thermal stability (ΔG_{unf}) of a three-state holoprotein (derivation of a Gibbs-Helmholtz-like equation).

Computational resources.

SUPPORTING INFORMATION TABLES

Tables S1-S4.

SUPPORTING INFORMATION FIGURES

Figures S1-S6.

SUPPORTING INFORMATION REFERENCES

Supporting Information Methods

Simulated Solvating Conditions. The protonation states set for the solution pH values simulated for the different proteins are shown in **Table S2**. Only the protonation states for histidine, aspartic acid, glutamic acid, N-ter amine and C-ter carboxyl residues are included. The pKa values used to set the protonation states are only indicated in non-trivial cases. Both the indicated and non-shown pKa values were obtained from the PDB2PQR server[1,2] or, in the case of WT CI2,[3] from NMR measurements. Given the pH values simulated, all basic residues (protonation and pKa values not shown in SI Table 2) were set to 'protonated'. Except for pseudo-lysozyme and nuclease (see footnote f in **Table S2**), salt concentration was set in the simulated systems to mimic the reported experimental ionic strength (see **Table 1** of the main text and **Table S2**). Those systems for which data reported under more than one experimental ionic strength were available, have been simulated either using one representative ionic strength value or the average. Ionic strengths have been modelled by adding the needed number of Na^+ and Cl^- ions after setting the equivalent salt concentration through the Gromacs[4] tool *gmx genion*. SI Table 2 also summarizes the protein charge, the number of water molecules and ions, and the box dimensions of the simulated systems. Importantly, solvating conditions for the unfolded ensembles (not shown in **Table S2**) are also adjusted to match the number of water molecules of their folded partner systems. Except for a small difference in the box dimensions, the solvating conditions for these unfolded ensembles are the same as those used for their folded counterparts (**Table S2**).

Calculation of thermal stability (ΔG_{unf}) of a three-state holoprotein (derivation of a Gibbs-Helmholtz-like equation). According to the following reaction scheme:

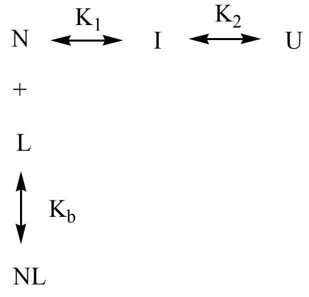

the unfolding equilibrium constant, K, of a holoprotein that binds a cofactor (or ligand) at a single binding site and whose apoprotein unfolds through a three-state mechanism (as it is the case of *Anabaena* holoFld) is:

$$K = \frac{[U]}{[N] + [NL]} = \frac{[U]}{[N] + K_b[N][L]} = \frac{[U]}{[N](1 + K_b[L])} = \frac{K_1 K_2}{1 + K_b[L]}$$

where K_1 and K_2 are the equilibrium constants of the first (native to intermediate) and second (intermediate to unfolded) unfolding steps, respectively; K_b is the binding constant of the complex formed between the native (N) protein and the cofactor or ligand (L), and the terms between brackets are the concentrations of free species in the equilibria.

Hence, the Gibbs free-energy change of the unfolding reaction can be written as:

$$\Delta G_{\text{holo(unf)}}(T) = -RT \cdot \ln(K_1K_2) + RT \cdot ln(1 + K_b(T) \cdot [L]) = \Delta G^0_{\text{apo(unf)}}(T) + RT \cdot ln(1 + K_b(T) \cdot [L]) = \Delta G^0_{\text{(Fol}\rightarrow\text{Int)}}(T) + \Delta G^0_{\text{(Int}\rightarrow\text{Unf)}}(T) + RT \cdot ln(1 + K_b(T) \cdot [L])$$

(1),

where $\Delta G^0_{\text{apo(unf)}}(T)$ in the case of *Anabaena* Fld is the overall stability of the three-state apoprotein, and $\Delta G^0_{\text{(Fol}\rightarrow\text{Int)}}(T)$ and $\Delta G^0_{\text{(Int}\rightarrow\text{Unf)}}(T)$ are the Gibbs free-energies of the partial unfolding steps, which can be described through the Gibbs-Helmholtz equation (**eq. 1** of the main text).

Wherever $K_b \gg 1/[L]$ **SI eq. 1** can be approximated by:

$$\Delta G_{\text{holo(unf)}}(T) = \Delta G^0_{\text{(Fol}\rightarrow\text{Int)}}(T) + \Delta G^0_{\text{(Int}\rightarrow\text{Unf)}}(T) + RT \cdot ln(K_b(T) \cdot [L]) \quad (2).$$

Binding constants are known to change with temperature[5] and the van't Hoff Equation[6] can be used to estimate their values from that at a reference temperature (e.g. 298.15 K):

$$ln\frac{K_b(T)}{K_b(298.15)} = \frac{-\Delta H_b + 298.15 \cdot \Delta Cp_b}{R} \cdot \left(\frac{1}{T} - \frac{1}{298.15}\right) + \frac{\Delta Cp_b}{R} ln\left(\frac{T}{298.15}\right) \quad (3).$$

Combining **SI eq. 2** and **SI eq. 3**:

$$\Delta G_{\text{holo(unf)}}(T) = \Delta G^0_{\text{(Fol}\rightarrow\text{Int)}}(T) + \Delta G^0_{\text{(Int}\rightarrow\text{Unf)}}(T) + RT \cdot \left\{lnK_b(298.15) + \frac{1}{R}\left[-\Delta H_b + 298.15 \cdot \Delta Cp_b \cdot \left(\frac{1}{T} - \frac{1}{298.15}\right) + \Delta Cp_b \cdot ln\left(\frac{T}{298.15}\right)\right]\right\} + RT \cdot ln[L]$$

(4),

which, at 1M of free ligand concentration becomes:

$$\Delta G_{\text{holo(unf)}}(T) = \Delta G^0_{\text{(Fol}\rightarrow\text{Int)}}(T) + \Delta G^0_{\text{(Int}\rightarrow\text{Unf)}}(T) + RT \cdot \left\{lnK_b(298.15) + \frac{1}{R}\left[-\Delta H_b + 298.15 \cdot \Delta Cp_b \cdot \left(\frac{1}{T} - \frac{1}{298.15}\right) + \Delta Cp_b \cdot ln\left(\frac{T}{298.15}\right)\right]\right\}$$

(5).

SI eq. S5 provides the temperature dependence of the standard Gibbs free-energy change of the holoprotein. It should be indicated that the van't Hoff equation cannot capture changes in the value of K_b due to changes in the conformation of the native protein that may take place as the temperature increases. Therefore, we will assume here that **SI eq. S5** may not hold above the first mid denaturation temperature of the apoprotein.

Computational resources. The sampling requirements of the protocol here presented are affordable with ordinary CPU- or GPU-clusters. The biggest, more challenging system here computed, 168-residue three-state apoFld (2.2 μs simulation: ~3.0 ns per 40 folded, 100 intermediate and 100 unfolded replicas, each at three temperatures), took 1-2 weeks of essentially unattended calculation on 32 cores (32 -OpenMP threads/1 MPI task) of an Intel Xeon E5-2680v3-based cluster (170 CPUs arranged in 85 nodes, https://cesar.unizar.es/hpc/). It is worth noting that this set of calculations could

likely be completed within a few hours (rough estimate) using a more powerful GPU-based computer cluster (e.g. ~50 GPUs), which is fairly common nowadays.

Supporting Information Tables

Table S1. MD setup broken down by simulation steps[a]

<table>
<tr><th>Simulation Step</th><th>General Settings (cutoffs)</th><th>PBC & Constraints</th><th>Step Setting</th><th>Thermodynamic Ensemble (baths)</th><th>Physical Conditions</th><th>Simulated Time</th></tr>
<tr><td>Minimization</td><td>Integrator: steepest descent,
Neighboring searching: grid,
rcoul (PME): 1.0 nm,
rvdw (cut-off): 1.0 nm</td><td>PBC: xyz,
Constraints: none</td><td>Emtol: 1.0 kJ/mol</td><td>-</td><td>Temp: 0 K
Press: 0 atm</td><td>max 20,000 steps</td></tr>
<tr><td>Volume adjustment[b]</td><td rowspan="6">Integrator: md,
Neighboring searching: grid,
rcoul (PME): 1.0 nm,
rvdw (cut-off): 1.0 nm,
vdw-modifier: potential-shift-verlet</td><td>PBC: xyz,
Constraints: all-bonds</td><td>t-step: 1 fs</td><td>NPT
Termost: v-rescale
Barost: Berendsen</td><td>Temp: 0 K
Press: 1 atm</td><td>100 ps</td></tr>
<tr><td>Heating</td><td rowspan="5">PBC: xyz,
Constraints: all-bonds</td><td>t-step: 1 fs</td><td>NVT,
Termost: Berendsen</td><td>Temp: Ini-T + ramp (6 x 50 K)
Press: 0 atm</td><td>7 x 50 ps</td></tr>
<tr><td>Equilibration 1</td><td>t-step: 1 fs</td><td>NVT,
Termost: v-rescale</td><td>Temp: Final-T
Press: 0 atm</td><td>150 ps</td></tr>
<tr><td>Equilibration 2</td><td rowspan="3">t-step: 2 fs</td><td>NPT,
Termost: v-rescale,
Barost: Berendsen</td><td rowspan="3">Temp: Final-T
Press: 1 atm</td><td>250 ps</td></tr>
<tr><td>Equilibration 3</td><td rowspan="2">NPT,
Termost: v-rescale,
Barost: Parrinello-Rahman</td><td>250 ps</td></tr>
<tr><td>Production</td><td>2 ns</td></tr>
<tr><td colspan="7" align="right">Total: 3.0 or 3.1 ns[c]</td></tr>
</table>

[a]MD setup done with Gromacs package (v2020)[4].

[b]MD step designed to re-accommodate the water molecules inside the solvating box after having removed a few of them so that they match in number those in the reference system(s) (see footnote h in **Table S2**).

[c]Total simulated time along all the steps. 3.1 ns were simulated when the 'volume-adjustment' step was needed. 3.0 ns were simulated otherwise.

Table S2. Solvating conditions and box dimensions in simulated systems[a]

System[b]	pH	pKa(s)[c]	Protonation State[d]	Protein Charge[e]	Ioinic Strength[f] (mM)	No. Ions[g]	No. Waters[h]	Box Diameter[i] (nm)	Box Volume (nm^3)
barnase	~4.1	Asp8:3.1 / Asp12:2.8 / Asp22:3.7 / Asp44:4.0 / Asp54:3.5 / Asp75:4.0 / Asp86:3.4 / Asp93:1.2 / Asp101:1.5 / Glu29:4.2 / Glu60:4.1 / Glu73:5.3 / His18:6.33 / His102:5.99 / N-ter:7.8 / C-ter:1.9	Glu^{prot} / His^{prot} / Asp^{unprot} / N-ter^{prot} / C-ter^{unprot}	+7	4.0	4 Na^+, 11 Cl^-	63,892	14	1,940
nuclease[j]	4.1	Asp19:7.5 / Asp21:3.4 / Asp40:5.2 / Asp82:3.9 / Asp95:3.7 / Asp144:3.6 / Asp146:3.6 / Glu10:4.1 / Glu43:3.2 / Glu52:4.3 / Glu57:4.6 / Glu67:3.6 / Glu73:4.7 / Glu75:4.3 / Glu122:4.6 / Glu129:5.6 / Glu135:4.2 / Glu142:4.6 / His8:6.4 / His46:6.0 / His121:5.7 / His124:5.9 / N-ter:7.7 / C-ter:3.2	Asp19^{prot} / Asp21^{unprot} / Asp40^{prot} / Asp82^{unprot} / Asp95^{unprot} / Asp144^{unprot} / Asp146^{unprot} / Glu10^{prot} / Glu43^{unprot} / Glu52^{prot} / Glu57^{prot} / Glu67^{unprot} / Glu73^{prot} / Glu75^{prot} / Glu122^{prot} / Glu129^{prot} / Glu135^{prot} / Glu142^{prot} / His8^{prot} / His46^{prot} / His121^{prot} / His124^{prot} / N-ter^{prot} / C-ter^{unprot}	+25	neut.	25 Cl^-	115,589	17	3,474
	5.0	pKas: idem to nuclease pH 4.1	Asp19^{prot}/ His ^{prot} / N-ter^{prot} / other Asp^{unprot} / Glu^{unprot} / C-ter^{unprot}	+14	neut.	14 Cl^-	115,602	17	3,474
	7.0	pKas: idem to nuclease pH 4.1	Asp19^{prot} / N-ter^{prot} / other Asp^{unprot} / Glu^{unprot} / His^{unprot}/ C-ter^{unprot}	+11	neut.	11 Cl^-	115,602	17	3,474

Table S2. Continuation…

System[b]	pH	pKa(s)[c]	Protonation State[d]	Protein Charge[e]	Ioinic Strength[f] (mM)	No. Ions[g]	No. Waters[h]	Box Diameter[i] (nm)	Box Volume (nm^3)
	7.0	pKas: idem to nuclease pH 4.1	Asp19[prot] / N-ter[prot] / other Asp[unprot] / Glu[unprot] / His[unprot]/ C-ter[unprot]	+11	neut.	11 Cl^-	115,602	17	3,474
WT CI2[k]	3.0	Glu24*:2.9±0.1 / Glu27*:2.9±0.1 / Glu34*:3.5±0.1 / Glu35*:2.9±0.1 / Asp43*:2.4 ±0.4 / Glu46*:3.7±0.1 / Glu61*:3.1±0.0 / Asp65*:3.6±0.1 / Asp72*:2.5±0.1 / Glu79:4.8 / N-ter:7.9 / C-ter:1.4	Glu24[unprot] / Glu27[unprot] / Glu34[prot] / Glu35[unprot] / Asp43[unprot] / Glu46[prot] / Glu61[prot] / Asp65[prot] / Asp72[unprot] / Glu79[prot] / N-ter[prot] / C-ter[unprot]	+5	1.9	1 Na^+, 6 Cl^-	30,950	11	941
	6.3	N-ter:7.9 / C-ter:1.4	Asp[unprot] / Glu[unprot] / N-ter[prot] / C-ter[unprot]	–1	33	19 Na^+, 18 Cl^-	30,902	11	941
Ile76Ala CI2[k]	3.0	pKas: idem to WT CI2 pH 3.0	Idem to WT CI2 pH 3.0	+5	1.9	1 Na^+, 6 Cl^-	30,950	11	941
WT lysozyme	2.4	Asp10:3.4 / Asp20:4.0 / Asp47:2.9 / Asp61:3.9 / Asp70:4.9 / Asp72:3.0 / Asp89:3.0 / Asp92:2.4 / Asp127:3.9 / Asp159:2.7 / Glu5:4.3 / Glu11:5.1 / Glu22:3.3 / Glu45:3.8 / Glu62:4.3 / Glu64:4.5 / Glu108:3.9 / Glu128:4.6 / His31:6.7 / N-ter:8.0 / C-ter:3.3	Asp[prot] / Glu[prot] / His[prot] / N-ter[prot] / C-ter[prot]	+28	34	71 Na^+, 99 Cl^-	115,371	17	3,474

Table S2. Continuation…

System[b]	pH	pKa(s)[c]	Protonation State[d]	Protein Charge[e]	Ioinic Strength[f] (mM)	No. Ions[g]	No. Waters[h]	Box Diameter[i] (nm)	Box Volume (nm^3)
Ile3Glu lysozyme	2.4	Glu3:3.7 / Other pKas: idem to WT lysozyme pH 2.4	Glu3^{prot} / Other Glu^{prot} / Asp^{prot} / His^{prot} / N-ter^{prot} / C-ter^{prot}	+28	34	71 Na^+, 99 Cl^-	115,371*	17,01*	vol. adj
pseudo-WT lysozyme[k]	3.0	Asp10:3.4 / Asp20:3.9 / Asp47:2.7 / Asp61:4.0 / Asp70:4.8 / Asp72:3.2 / Asp89:2.8 / Asp92:2.1 / Asp127:3.9 / Asp159:2.6 / Glu5:4.4 / Glu11:5.0 / Glu22:3.3 / Glu45:3.7 / Glu62:2.8 / Glu64:4.7 / Glu108:3.9 / Glu128:4.6 / His31:6.6 / N-ter:8.0 / C-ter:3.3	Asp10^{prot} / Asp20^{prot} / Asp47^{unprot} / Asp61^{prot} / Asp70^{prot} / Asp72^{prot} / Asp89^{unprot} / Asp92^{unprot} / Asp127^{prot} / Asp159^{unprot} / Glu5^{prot} / Glu11^{prot} / Glu22^{prot} / Glu45^{prot} / Glu62^{unprot} / Glu64^{prot} / Glu108prot / Glu128^{prot} / His31^{prot} / N-ter^{prot} / C-ter^{prot}	+23	neut.	23 Cl^-	115,531	17	3,474
	3.7	pKas: idem to pseudo-WT lysozyme pH 3.0	Asp10^{unprot} / Asp72^{unprot} / Glu22^{unprot} / N-ter^{prot} / C-ter^{unprot} / other residues: idem to pseudo-WT lysozyme pH 3.0	+19	neut.	19 Cl^-	115,535	17	3,474

Table S2. Continuation…

System[b]	pH	pKa(s)[c]	Protonation State[d]	Protein Charge[e]	Ioinic Strength[f] (mM)	No. Ions[g]	No. Waters[h]	Box Diameter[i] (nm)	Box Volume (nm^3)
apoFld†	7.0	His35:5.4 / N-ter:7.2 / C-ter:3.4	His35^{unprot} / Asp^{unprot} / Glu^{unprot} / N-ter^{prot} / C-ter^{unprot}	–17	17	51 Na^+, 34 Cl^-	136,732	18	4,124
apoFld† (intermediate)	7.0	His35:5.5 / N-ter:7.7 / C-ter:3.3	His35^{unprot} / Asp^{unprot} / Glu^{unprot} / N-ter^{prot} / C-ter^{unprot}	–17	17	51 Na^+, 34 Cl^-	136,732*	19*	vol. adj
holoFld	7.0	His35:5.3 / N-ter:7.3 / C-ter:3.3	His35^{unprot} / Asp^{unprot} / Glu^{unprot} / N-ter^{prot} / C-ter^{unprot}	–19	17	53 Na^+, 34 Cl^-	145,423	19	4,524
apoFld + FMN	7.0	His35:5.4 / N-ter:7.2 / C-ter:3.4	His35^{unprot} / Asp^{unprot} / Glu^{unprot} / N-ter^{prot} / C-ter^{unprot}	–19	17	53 Na^+, 34 Cl^-	145,423*	20*	vol. adj

[a]Solvating conditions for systems simulated using Charmm22-CMAP and Tip3p. Same conditions are used to simulate folded and unfolded systems, and for systems simulated with Amber99SB-ILDN and Tip3p. **Note:** in order to have the same charge and, therefore, the same number of ions and water molecules in folded and unfolded systems, identical pKa values are assumed for the folded and the unfolded state even if it is not strictly the case.

[b]Proteins or cofactors (FMN) simulated in this work. (†): systems which have been additionally simulated with Amber99SB-ILDN.

[c]pKa values for the native protein (folded) either obtained from the PDB2PQR (PROPKA 3.0[7]) server[1,2] (no errors given) or reported by Tan *et al.*[3] (indicated with an asterisk (*) next to the residue name).

[d]Protonation states resulted from the simulated pH and pKa values (equal for folded and unfolded states, see the Note in footnote a).

[e]Protein charge after setting protonation.

[f]Ionic strength (IS) established in the systems (by adding Na^+ and Cl^- ions) based on the values used in the corresponding experimental determination of the thermal unfolding parameters. For pseudo-lysozyme, the experimental IS (see Table 1) are lower than those obtained after neutralizing the system prior to simulation so no extra ions have been added after neutralization. For nuclease, the unfolding thermodynamics measurements have been described to be independent of IS[8] and the experimental ISs used in the reference experimental works vary in a large range, so no extra ions have been added after neutralization. Individual and averaged ISs used in the

experimental measurements along with the papers of reference are given in **Table 1** of the main text.

[g]Number of ions added by Gromacs *gmx genion* tool after setting the required salt concentration.

[h]Number of water molecules finally simulated in the systems. (*): systems where the number of water molecules was initially higher (when setting the box diameter) but it was reduced to match the number of waters in the corresponding reference system(s). Example: to calculate the unfolding energetics of holoFld, two systems were involved: 1) boxes with folded holoFld and 2) boxes with unfolded Fld (apo) plus FMN unbound (see **Methods** and **Figure 4a** in the manuscript). In this case, the number of water molecules in the systems with unfolded apoFld plus FMN is initially set bigger than the numbers of this molecule previously settled in the folded holoFld system (the reference here). Then, as required in this protocol (based on calculation by difference of thermodynamics of unfolding states) a few water molecules have to be removed to equate their number in the reference system. In these cases, a 'volume-adjustment' step (indicated as 'vol. adj.' in the 'Box volume' column) is introduced in the preparation phase before the heating step (**Table S1**).

[i]Truncated rhombic dodecahedron boxes. Dimensions defined in each case by the unfolded structure with maximum diameter among those in the filtered unfolded ensemble: a minimum distance of 1 nm was left between the farthest atom of the unfolded protein from the center and the box edges. (*): Initial diameter settled before removing a few water molecules and adjusting the box volume.

[j]83-231 (renumbered 1-149) C-ter fragment of nuclease as in PDB ID 2SNS (see **Methods** in the main text).

[k]Truncated CI2 structures lacking the first 19 amino acid residues.

[l]Lysozyme variant wherein cysteine residues at positions 54 and 97 are replaced by a threonine and an alanine, respectively.

Table S3. Radius of gyration (Rg) at selected times over the simulated 2-ns trajectories, and statistical comparison

Protein (pH)[a]	Force field / FMN-Parameterization[b]	State[c]	Temp[d] (K)	Rg[0e] (nm)	Rg[f] ($t_{0/4}$) (nm)	Rg[f] ($t_{1/4}$) (nm)	Rg[f] ($t_{2/4}$) (nm)	Rg[f] ($t_{3/4}$) (nm)	Rg[f] ($t_{4/4}$) (nm)	P-value[g]
barnase	Charmm22-CMAP	folded	295	1.351	1.366	1.368	1.371	1.372	1.371	0.136
barnase	Charmm22-CMAP	folded	315	1.351	1.371	1.370	1.373	1.370	1.373	0.683
barnase	Charmm22-CMAP	folded	335	1.351	1.370	1.370	1.370	1.368	1.368	0.903
barnase	Charmm22-CMAP	unfolded	295	2.655	2.627	2.584	2.613	2.599	2.599	0.999
barnase	Charmm22-CMAP	unfolded	315	2.655	2.658	2.620	2.646	2.571	2.533	0.922
barnase	Charmm22-CMAP	unfolded	335	2.655	2.631	2.608	2.570	2.449	2.397	0.517
nuclease (pH 4.1)	Charmm22-CMAP	folded	307	1.540	1.555	1.557	1.557	1.561	1.567	0.291
nuclease (pH 4.1)	Charmm22-CMAP	folded	317	1.540	1.560	1.563	1.564	1.570	1.569	0.339
nuclease (pH 4.1)	Charmm22-CMAP	folded	327	1.540	1.565	1.566	1.569	1.565	1.565	0.961
nuclease (pH 4.1)	Charmm22-CMAP	unfolded	307	3.379	3.445	3.509	3.497	3.511	3.499	0.998
nuclease (pH 4.1)	Charmm22-CMAP	unfolded	317	3.379	3.593	3.614	3.674	3.664	3.637	0.989
nuclease (pH 4.1)	Charmm22-CMAP	unfolded	327	3.379	3.530	3.638	3.594	3.631	3.695	0.936
nuclease (pH 5.0)	Charmm22-CMAP	folded	307	1.540	1.572	1.580	1.575	1.569	1.566	0.572
nuclease (pH 5.0)	Charmm22-CMAP	folded	317	1.540	1.566	1.564	1.566	1.560	1.555	0.521
nuclease (pH 5.0)	Charmm22-CMAP	folded	327	1.540	1.578	1.565	1.563	1.553	1.554	0.009
nuclease (pH 5.0)	Charmm22-CMAP	unfolded	307	3.286	3.171	3.133	3.088	3.085	3.124	0.984
nuclease (pH 5.0)	Charmm22-CMAP	unfolded	317	3.286	3.165	3.154	3.134	3.182	3.090	0.986
nuclease (pH 5.0)	Charmm22-CMAP	unfolded	327	3.286	3.123	3.150	3.122	3.028	2.937	0.695

Table S3. Continuation…

**Protein (pH)**[a]	**Force field / FMN-parameterization**[b]	**State**[c]	**Temp**[d] **(K)**	**Rg**[0e] **(nm)**	**Rg**[f] **($t_{0/4}$) (nm)**	**Rg**[f] **($t_{1/4}$) (nm)**	**Rg**[f] **($t_{2/4}$) (nm)**	**Rg**[f] **($t_{3/4}$) (nm)**	**Rg**[f] **($t_{4/4}$) (nm)**	**P-value**[g]
nuclease (pH 7.0)	Charmm22-CMAP	folded	307	1.540	1.555	1.548	1.553	1.556	1.550	0.575
nuclease (pH 7.0)	Charmm22-CMAP	folded	317	1.540	1.563	1.560	1.554	1.552	1.550	0.219
nuclease (pH 7.0)	Charmm22-CMAP	folded	327	1.540	1.561	1.558	1.563	1.557	1.552	0.615
nuclease (pH 7.0)	Charmm22-CMAP	unfolded	307	3.379	3.381	3.396	3.406	3.396	3.366	1.000
nuclease (pH 7.0)	Charmm22-CMAP	unfolded	317	3.379	3.487	3.450	3.392	3.382	3.346	0.944
nuclease (pH 7.0)	Charmm22-CMAP	unfolded	327	3.379	3.448	3.457	3.448	3.435	3.382	0.994
WT CI2 (pH 3.0)	Charmm22-CMAP	folded	320	1.129	1.138	1.139	1.137	1.137	1.140	0.311
WT CI2 (pH 3.0)	Charmm22-CMAP	folded	335	1.129	1.136	1.141	1.137	1.137	1.136	0.124
WT CI2 (pH 3.0)	Charmm22-CMAP	folded	350	1.129	1.142	1.140	1.138	1.137	1.137	0.206
WT CI2 (pH 3.0)	Charmm22-CMAP	unfolded	320	2.251	2.303	2.199	2.190	2.146	2.150	0.827
WT CI2 (pH 3.0)	Charmm22-CMAP	unfolded	335	2.251	2.100	2.031	1.964	1.910	1.924	0.521
WT CI2 (pH 3.0)	Charmm22-CMAP	unfolded	350	2.251	2.054	2.050	1.987	1.958	1.921	0.809
WT CI2 (pH 6.3)	Charmm22-CMAP	folded	335	1.129	1.134	1.134	1.131	1.130	1.130	0.072
WT CI2 (pH 6.3)	Charmm22-CMAP	folded	350	1.129	1.134	1.131	1.130	1.131	1.125	0.010
WT CI2 (pH 6.3)	Charmm22-CMAP	folded	365	1.129	1.130	1.131	1.129	1.133	1.130	0.461
WT CI2 (pH 6.3)	Charmm22-CMAP	unfolded	335	2.251	2.273	2.215	2.100	2.080	2.002	0.391
WT CI2 (pH 6.3)	Charmm22-CMAP	unfolded	350	2.251	2.049	1.900	1.818	1.802	1.806	0.358
WT CI2 (pH 6.3)	Charmm22-CMAP	unfolded	365	2.251	1.938	1.966	1.903	1.848	1.740	0.339

Table S3. Continuation…

Protein (pH)[a]	Force field / FMN-parameterization[b]	State[c]	Temp[d] (K)	Rg[0e] (nm)	Rg[f] ($t_{0/4}$) (nm)	Rg[f] ($t_{1/4}$) (nm)	Rg[f] ($t_{2/4}$) (nm)	Rg[f] ($t_{3/4}$) (nm)	Rg[f] ($t_{4/4}$) (nm)	P-value[g]
Ile76-Ala CI2 (pH 3.0)	Charmm22-CMAP	folded	320	1.131	1.136	1.136	1.136	1.135	1.134	0.858
Ile76-Ala CI2 (pH 3.0)	Charmm22-CMAP	folded	335	1.131	1.136	1.136	1.135	1.136	1.136	0.964
Ile76-Ala CI2 (pH 3.0)	Charmm22-CMAP	folded	350	1.131	1.139	1.138	1.135	1.137	1.136	0.542
Ile76-Ala CI2 (pH 3.0)	Charmm22-CMAP	unfolded	320	2.309	2.149	2.110	2.099	2.107	2.103	0.994
Ile76-Ala CI2 (pH 3.0)	Charmm22-CMAP	unfolded	335	2.309	2.099	2.052	1.980	1.897	1.829	0.217
Ile76-Ala CI2 (pH 3.0)	Charmm22-CMAP	unfolded	350	2.309	2.052	2.035	2.032	1.944	1.922	0.852
WT lysozyme (pH 2.4)	Charmm22-CMAP	folded	305	1.652	1.702	1.702	1.704	1.696	1.697	0.656
WT lysozyme (pH 2.4)	Charmm22-CMAP	folded	320	1.652	1.708	1.706	1.711	1.697	1.701	0.299
WT lysozyme (pH 2.4)	Charmm22-CMAP	folded	335	1.652	1.707	1.706	1.708	1.711	1.699	0.698
WT lysozyme (pH 2.4)	Charmm22-CMAP	unfolded	305	3.644	3.442	3.473	3.474	3.491	3.432	0.998
WT lysozyme (pH 2.4)	Charmm22-CMAP	unfolded	320	3.644	3.438	3.449	3.422	3.470	3.513	0.993
WT lysozyme (pH 2.4)	Charmm22-CMAP	unfolded	335	3.644	3.413	3.428	3.477	3.419	3.403	0.991
Ile3Glu lysozyme (pH 2.4)	Charmm22-CMAP	folded	305	1.652	1.700	1.702	1.710	1.706	1.703	0.563
Ile3Glu lysozyme (pH 2.4)	Charmm22-CMAP	folded	320	1.652	1.705	1.708	1.707	1.705	1.710	0.972
Ile3Glu lysozyme (pH 2.4)	Charmm22-CMAP	folded	335	1.652	1.707	1.703	1.700	1.706	1.704	0.940
Ile3Glu lysozyme (pH 2.4)	Charmm22-CMAP	unfolded	305	3.700	3.538	3.551	3.593	3.602	3.610	0.994
Ile3Glu lysozyme (pH 2.4)	Charmm22-CMAP	unfolded	320	3.700	3.263	3.195	3.277	3.324	3.369	0.917
Ile3Glu lysozyme (pH 2.4)	Charmm22-CMAP	unfolded	335	3.700	3.310	3.264	3.323	3.315	3.281	0.998

Table S3. Continuation…

Protein (pH)[a]	Force field / FMN-parameterization[b]	State[c]	Temp[d] (K)	Rg[0e] (nm)	Rg[f] ($t_{0/4}$) (nm)	Rg[f] ($t_{1/4}$) (nm)	Rg[f] ($t_{2/4}$) (nm)	Rg[f] ($t_{3/4}$) (nm)	Rg[f] ($t_{4/4}$) (nm)	P-value[g]
pseudo-WT lysozyme (pH 3.0)	Charmm22-CMAP	folded	305	1.651	1.707	1.706	1.704	1.703	1.707	0.987
pseudo-WT lysozyme (pH 3.0)	Charmm22-CMAP	folded	320	1.651	1.704	1.710	1.709	1.717	1.722	0.253
pseudo-WT lysozyme (pH 3.0)	Charmm22-CMAP	folded	335	1.651	1.711	1.709	1.717	1.711	1.714	0.963
pseudo-WT lysozyme (pH 3.0)	Charmm22-CMAP	unfolded	305	3.602	3.351	3.356	3.416	3.440	3.449	0.974
pseudo-WT lysozyme (pH 3.0)	Charmm22-CMAP	unfolded	320	3.602	3.317	3.316	3.319	3.305	3.301	1.000
pseudo-WT lysozyme (pH 3.0)	Charmm22-CMAP	unfolded	335	3.602	3.345	3.341	3.318	3.333	3.326	1.000
pseudo-WT lysozyme (pH 3.7)	Charmm22-CMAP	folded	305	1.651	1.665	1.671	1.665	1.664	1.666	0.897
pseudo-WT lysozyme (pH 3.7)	Charmm22-CMAP	folded	320	1.651	1.668	1.663	1.665	1.671	1.672	0.718
pseudo-WT lysozyme (pH 3.7)	Charmm22-CMAP	folded	335	1.651	1.662	1.672	1.674	1.681	1.680	0.099
pseudo-WT lysozyme (pH 3.7)	Charmm22-CMAP	unfolded	305	3.602	3.341	3.285	3.252	3.285	3.328	0.988
pseudo-WT lysozyme (pH 3.7)	Charmm22-CMAP	unfolded	320	3.602	3.259	3.253	3.240	3.305	3.324	0.983
pseudo-WT lysozyme (pH 3.7)	Charmm22-CMAP	unfolded	335	3.602	3.299	3.298	3.289	3.234	3.227	0.989
apoFld	Charmm22-CMAP	folded	305	1.465	1.486	1.486	1.487	1.488	1.489	0.126
apoFld	Charmm22-CMAP	folded	320	1.465	1.488	1.490	1.491	1.491	1.490	0.168
apoFld	Charmm22-CMAP	folded	335	1.465	1.492	1.493	1.493	1.492	1.494	0.849
apoFld	Charmm22-CMAP	intermediate	305	1.956	1.863	1.858	1.849	1.840	1.824	0.712
apoFld	Charmm22-CMAP	intermediate	320	1.956	1.867	1.839	1.828	1.802	1.803	0.042[h]
apoFld	Charmm22-CMAP	intermediate	335	1.956	1.835	1.783	1.764	1.743	1.743	0.000[h]

Table S3. Continuation…

Protein (pH)[a]	Force field / FMN-Parameterization[b]	State[c]	Temp[d] (K)	Rg^{0e} (nm)	Rg^f ($t_{0/4}$) (nm)	Rg^f ($t_{1/4}$) (nm)	Rg^f ($t_{2/4}$) (nm)	Rg^f ($t_{3/4}$) (nm)	Rg^f ($t_{4/4}$) (nm)	P-value[g]
apoFld	Charmm22-CMAP	unfolded	305	3.522	3.412	3.378	3.340	3.236	3.150	0.833
apoFld	Charmm22-CMAP	unfolded	320	3.522	3.365	3.332	3.315	3.265	3.099	0.712
apoFld	Charmm22-CMAP	unfolded	335	3.522	3.254	3.294	3.242	3.183	3.051	0.823
apoFld	Amber99SB-ILDN	folded	305	1.465	1.481	1.478	1.480	1.479	1.481	0.443
apoFld	Amber99SB-ILDN	folded	320	1.465	1.482	1.481	1.480	1.481	1.482	0.854
apoFld	Amber99SB-ILDN	folded	335	1.465	1.480	1.482	1.481	1.481	1.482	0.941
apoFld	Amber99SB-ILDN	intermediate	305	1.956	1.879	1.858	1.843	1.842	1.832	0.266
apoFld	Amber99SB-ILDN	intermediate	320	1.956	1.852	1.849	1.856	1.837	1.834	0.875
apoFld	Amber99SB-ILDN	intermediate	335	1.956	1.869	1.839	1.835	1.815	1.806	0.050[h]
apoFld	Amber99SB-ILDN	unfolded	305	3.526	3.559	3.544	3.588	3.578	3.623	0.999
apoFld	Amber99SB-ILDN	unfolded	320	3.526	3.276	3.256	3.244	3.240	3.232	1.000
apoFld	Amber99SB-ILDN	unfolded	335	3.526	3.290	3.275	3.290	3.332	3.281	0.999
holoFld	Charmm22-CMAP/Par.-1	folded	305	1.460	1.485	1.485	1.485	1.484	1.486	0.817
holoFld	Charmm22-CMAP/Par.-1	folded	320	1.460	1.485	1.486	1.486	1.485	1.488	0.361
holoFld	Charmm22-CMAP/Par.-1	folded	335	1.460	1.490	1.489	1.489	1.489	1.489	0.930
holoFld	Charmm22-CMAP/Par.-2	folded	305	0.457	0.461	0.460	0.459	0.460	0.459	0.222
holoFld	Charmm22-CMAP/Par.-2	folded	320	0.457	0.461	0.460	0.460	0.460	0.461	0.924
holoFld	Charmm22-CMAP/Par.-2	folded	335	0.457	0.459	0.460	0.460	0.459	0.460	0.833
holoFld	Charmm22-CMAP/Par.-3	folded	305	1.460	1.486	1.484	1.485	1.483	1.485	0.742
holoFld	Charmm22-CMAP/Par.-3	folded	320	1.460	1.486	1.486	1.485	1.488	1.484	0.312
holoFld	Charmm22-CMAP/Par.-3	folded	335	1.460	1.488	1.486	1.488	1.486	1.489	0.592

[a]Protein or variant simulated. When a protein has been simulated at several pH values, the pH of the simulation is indicated between parentheses (see**Table S2**).

[b]Force field used in the simulation. In simulations of holoFld the FMN parameterization used is indicated (see section **Methods** of the main text).

[c]Folding state (folded, intermediate or unfolded) of the simulated systems.

[d]Temperature at which the trajectories where the Rg is analyzed were run.

[e]Radius of gyration of the crystal structure (see PDB IDs in the main text), average Rg of the initial structures selected from the unfolded ensembles generated by ProtSA[9], or average Rg of the NMR ensemble (PDB 2KQU[10]) representing the apoFld intermediate state.

[f]Average radius of gyration of the proteins (for all the simulated replicas per system) over time in the 2ns-production trajectories run, namely: $t_{0/4}$ at the beginning of the trajectory (t=0 ns), $t_{1/4}$ at the end of the first quartile of the trajectory (t=0.5 ns), $t_{2/4}$ at the middle of the trajectory (t=1.0 ns), $t_{3/4}$ at the end of the third quartile of the trajectory (t=1.5 ns), and $t_{4/4}$ at the end of the trajectory (t=2.0 ns).

[g]P-value of an ANOVA statistical test performed to compare the protein Rg means at the checkpoints indicated. Analysis done for assessing the Rg evolution over time in relation with the known issue of structure overcompaction reported[11,12] for force fields like Charmm22-CMAP[13].

[h]Significant P-values (for a 95 % of confidence) indicated. These values only appear in some systems of the apoFld intermediate state. In this state, the apoFld structure simulated (PDB 2KQU[10]) presents disordered regions –appearing quite stretched in the NMR models− that slightly shrink at the beginning of the simulation towards the central structured nucleus albeit without forming new secondary structures nor compacting the already folded structure of the protein.

Table S4. Time-averaged enthalpies (⟨H_{state}⟩ ± standard error (SE)) per system and simulated temperature. Values used in the protocol to calculate by difference the enthalpy change upon unfolding (ΔH_{unf})

		barnase kcal/mol (±SE)	nuclease kcal/mol (±SE)			CI2 kcal/mol (±SE)		
**State**	**Temp[a]**	**WT pH ~4.1**	**WT pH 4.1**	**WT pH 5.0**	**WT pH 7.0**	**WT pH 3.0**	**WT pH 6.3**	**Ile76Ala pH 3.0**
Folded	T1	−506,461 (±1)	−887,731 (±3)	−887,492 (±2)	−887,120 (±3)	−230,686 (±1)	−226,942 (±1)	−230,697 (±1)
	T2	−482,056 (±1)	−865,762 (±4)	−865,523 (±2)	−865,151 (±4)	−221,874 (±1)	−218,164 (±1)	−221,885 (±1)
	T3	−457,835 (±2)	−843,875 (±3)	−843,630 (±2)	−843,259 (±3)	−213,081 (±1)	−209,383 (±1)	−213,096 (±1)
Unfolded	T1	−506,381 (±1)	−887,659 (±1)	−887,441 (±1)	−887,079 (±2)	−230,648 (±1)	−226,897 (±1)	−230,665 (±1)
	T2	−481,951 (±1)	−865,672 (±1)	−865,452 (±2)	−865,090 (±1)	−221,828 (±1)	−218,111 (±1)	−221,845 (±1)
	T3	−457,714 (±1)	−843,769 (±1)	−843,544 (±2)	−843,185 (±1)	−213,030 (±1)	−209,324 (±1)	−213,048 (±1)

Table S4. Continuation…

		lysozyme kcal/mol (±SE)		pseudo-lysozyme kcal/mol (±SE)	
**State**	**Temp[a]**	**WT pH 2.4**	**Ile3Glu pH 2.4**	**WT* pH 3.0**	**WT* pH 3.7**
Folded	T1	−906,054 (±2)	−906,103 (±2)	−893,020 (±2)	−892,948 (±1)
	T2	−873,131 (±1)	−873,186 (±1)	−860,068 (±2)	−860,007 (±2)
	T3	−840,396 (±2)	−840,452 (±2)	−827,300 (±2)	−827,228 (±2)
Unfolded	T1	−905,884 (±1)	−905,940 (±1)	−892,847 (±1)	−892,780 (±2)
	T2	−872,945 (±1)	−873,002 (±1)	−859,878 (±2)	−859,804 (±2)
	T3	−840,186 (±1)	−840,243 (±1)	−827,081 (±2)	−827,008 (±1)

Table S4. Continuation…

		apoFld kcal/mol (±SE)		holoFld kcal/mol (±SE)		
**State**	**Temp**[a]	**Charmm22-CMAP**	**Amber99SB-ILDN**	**Charmm22-CMAP/Par.-1**	**Charmm22-CMAP/Par.-2**	**Charmm22-CMAP/Par.-3**
Folded	T1	−1,055,434 (±4)	−1,055,875 (±3)	−1,122,685 (±3)	−1,122,628 (±3)	−1,122,702 (±4)
	T2	−1,016,485 (±4)	−1,016,930 (±2)	−1,081,263 (±5)	−1,081,206 (±3)	−1,081,290 (±3)
	T3	−977,741 (±4)	−978,187 (±4)	−1,040,057 (±5)	−1,039,997 (±3)	−1,040,081 (±4)
Unfolded	T1	−1,055,390 (±1)	−1,055,849 (±2)	−1,122,677 (±3)[b]	−1,122,622 (±3)[b]	−1,122,692 (±3)[b]
	T2	−1,016,406 (±1)	−1,016,867 (±2)	−1,081,214 (±4)[b]	−1,081,173 (±3)[b]	−1,081,239 (±3)[b]
	T3	−977,622 (±1)	−0,978,086 (±2)	−1,039,958 (±4)[b]	−1,039,904 (±3)[b]	−1,039,994 (±3)[b]
Intermediate	T1	−1,055,414 (±2)	−1,055,840 (±2)			
	T2	−1,016,445 (±2)	−1,016,877 (±2)			
	T3	−977,676 (±2)	−978,111 (±2)			

[a]Simulated temperatures for each system appear in **Table 2** of the main text. Since they are different for each simulated system, they are only listed here as T1, T2 and T3 for the sake of space.

[b]Refers to systems where the unfolded apoprotein and one unbound FMN molecule are simulated in the same box (see **Methods** and **Figure 4a** of the main text).

Supporting Information Figures

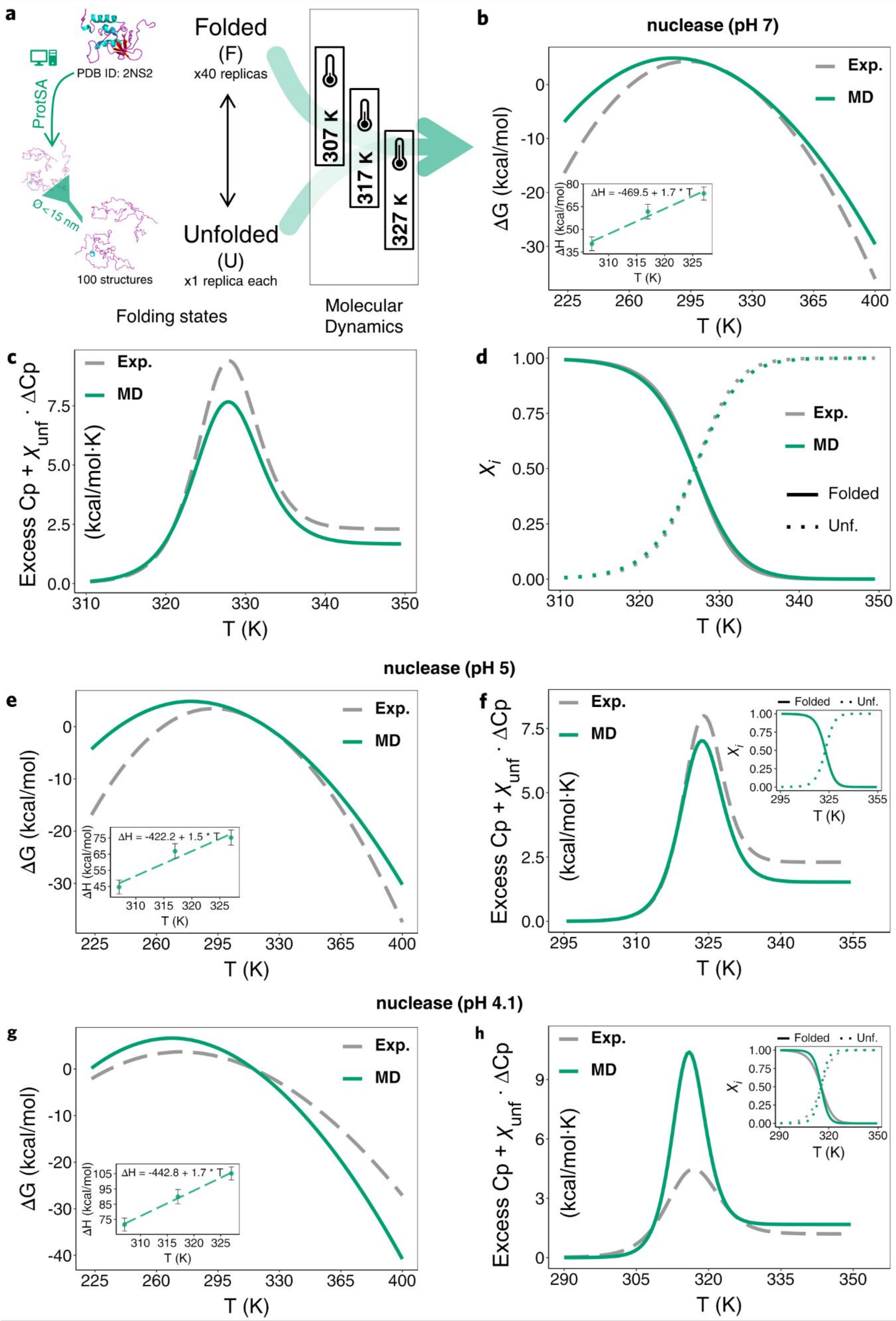

Figure S1. Simplified MD-based scheme and comparison with experimental results for nuclease. a) Protein models and folding states, number of structures (unfolded) and replicas (folded) simulated, diameter cutoff used to filter too-elongated unfolded structures obtained from ProtSA[25] (left, see also **Figure S5**), and temperatures selected for the MD-based calculation (Charmm22-CMAP) of nuclease thermodynamics at pH 7.0, 5.0 and 4.1. **b-d)** Global stability curves ($\Delta G_{unf}(T)$), thermograms (Excess Cp + χ_{unf}.× ΔCp vs. T), and protein molar fractions (χ_i) vs. T (computed vs. experimental), respectively, obtained for nuclease simulated at pH 7.0. **e** and **f)** Global stability curves and thermograms (computed vs. experimental), respectively, obtained for nuclease simulated at pH 5.0. **g** and **h)** Global stability curves and thermograms (computed vs. experimental), respectively, obtained for nuclease simulated at pH 4.1. The ΔH_{unf} vs. T linear plot for pH ~5.0 (and ~4.1) appears as an inset in **e** (and **g**). The protein species fraction (χ_i) vs. T plot for pH 5.0 (and 4.1) is included as an inset in **f** (and **h**). The colour-coding is indicated in the legends of the panels.

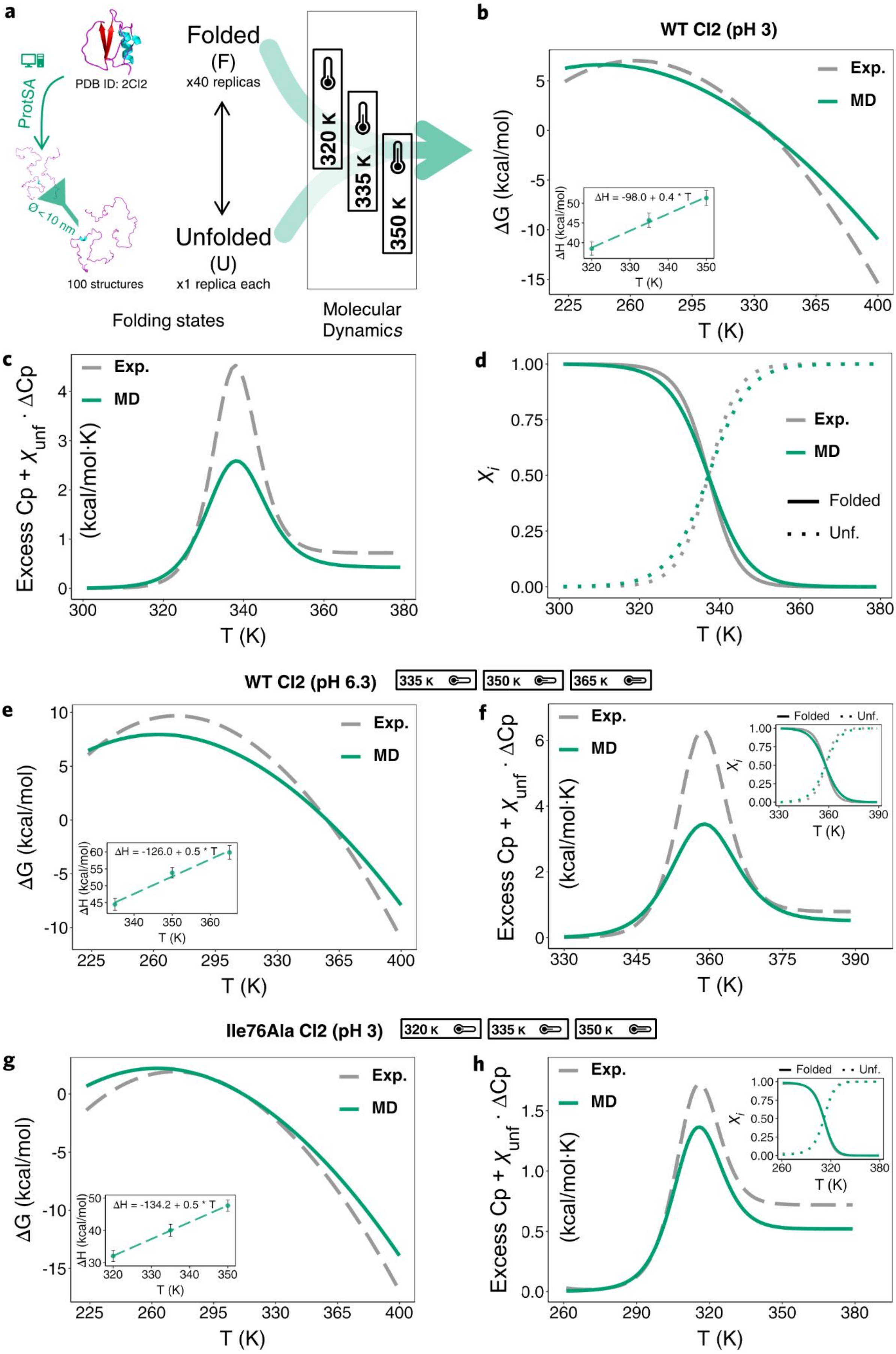

a
PDB ID: 2CI2
ProtSA
Ø<10 nm
100 structures
Folded
(F)
x40 replicas
Unfolded
(U)
x1 replica each
Folding states
320 K
335 K
350 K
Molecular Dynamics
b
WT CI2 (pH 3)
Exp.
MD
ΔG (kcal/mol)
T (K)
ΔH = -98.0 + 0.4 * T
ΔH (kcal/mol)
c
Excess Cp + X_unf · ΔCp (kcal/mol·K)
d
X_i
Folded
Unf.
WT CI2 (pH 6.3)
335 K
350 K
365 K
e
ΔH = -126.0 + 0.5 * T
f
Ile76Ala CI2 (pH 3)
320 K
335 K
350 K
g
ΔH = -134.2 + 0.5 * T
h

Figure S2. Simplified MD-based scheme and comparison with experimental results for CI2. a) Protein models and folding states, number of structures (unfolded) and replicas (folded) simulated, diameter cutoff used to filter too-elongated unfolded structures obtained from ProtSA[25] (left, see also **Figure S5**), and temperatures selected for the MD-based calculation (Charmm22-CMAP) of CI2 thermodynamics. **b-d)** Global stability curves ($\Delta G_{unf}(T)$), thermograms (Excess Cp + χ_{unf}.× ΔCp vs. T), and protein molar fractions (χ_i) vs. T, respectively (*in silico* vs. experimental), obtained for WT CI2 at pH 3.0. Inset in **b** depicts the ΔH_{unf} vs. T linear plot with the fitted equation (the slope being ΔCp_{unf}) obtained from the MD simulations. **e** and **f)** Global stability curves and thermograms (computed vs. experimental), respectively, obtained for WT CI2 at pH 6.3. The ΔH_{unf} vs. T linear plot appears as an inset in **e** and the protein species fraction (χ_i) vs. T plot is included as an inset in **f**. **g** and **h**) Global stability curves and thermograms (computed vs. experimental), respectively, obtained for Ile76Ala at pH 3.0. Insets in **g** and **h** similar to those in **e** and **f**, respectively. The colour-coding is indicated in the legends of the panels.

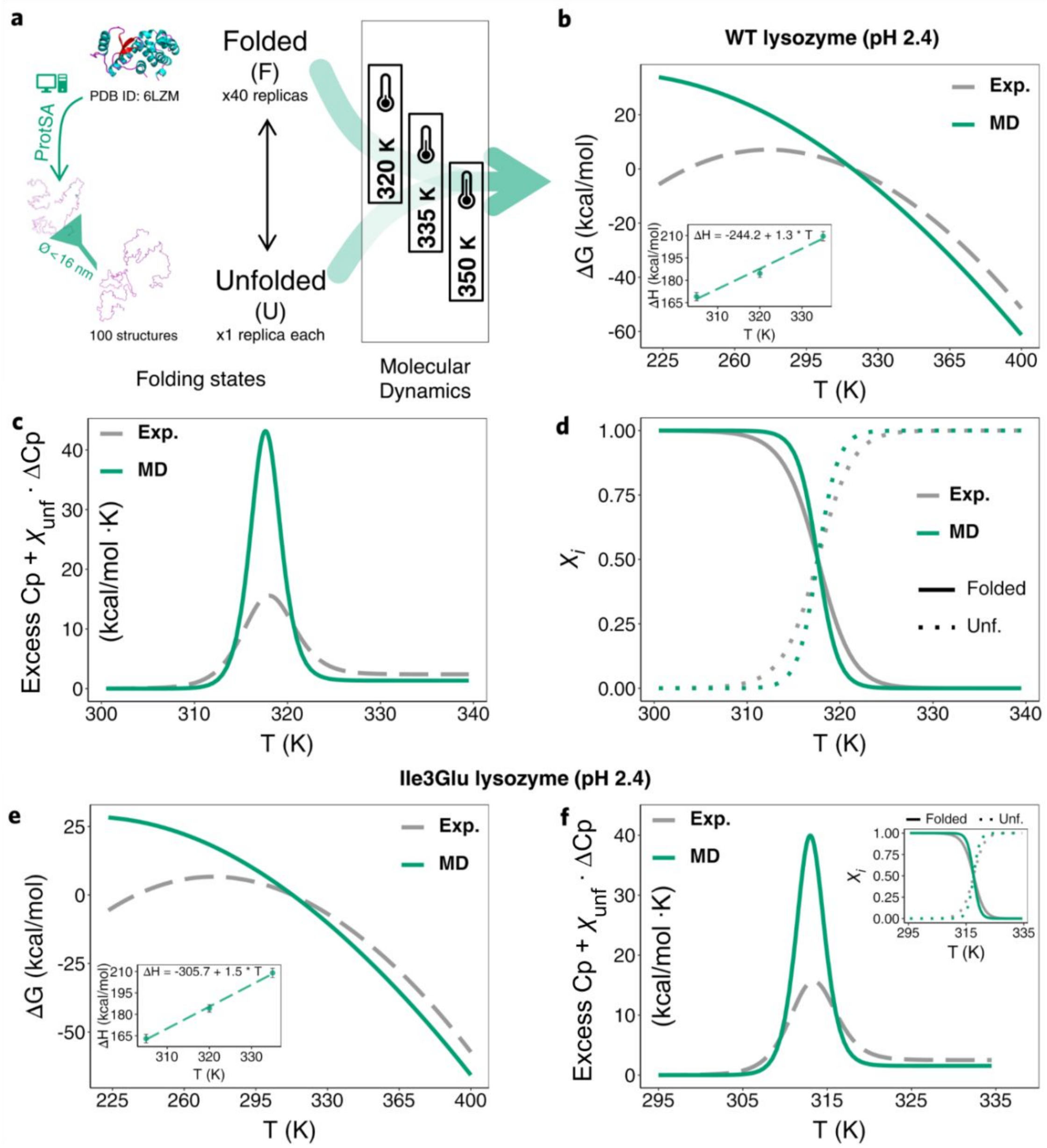

Figure S3. Simplified MD-based scheme and comparison with experimental results for lysozyme. a) The protein models and folding states, the number of structures (unfolded) and replicas (folded) simulated, the diameter cutoff used to filter too-elongated unfolded structures obtained from ProtSA[25] (left, see also **Figure S5**), and temperatures selected for the MD-based calculation (Charmm22-CMAP) of thermodynamics of WT lysozyme and the variant Ile3Glu at pH 2.4. **b-d)** Global stability curves ($\Delta G_{unf}(T)$), thermograms (Excess Cp + χ_{unf}.× ΔCp vs. T), and protein molar fractions (χ_i) vs. T (*in silico* vs. experimental), respectively, obtained for WT lysozyme at pH 2.4. Inset in **b** depicts the ΔH_{unf} vs. T linear plot with the fitted equation (the slope being ΔCp_{unf}) obtained from the MD simulations. **e** and **f)** Global stability curves ($\Delta G_{unf}(T)$) and thermograms (in silico vs. experimental), respectively, obtained for Ile3Glu lysozyme at pH 2.4. Inset in **e** similar to that in **b**. The protein species fraction (χ_i) vs. T plot is included as an inset in **f**. The colour-coding is indicated in the legends of the panels.

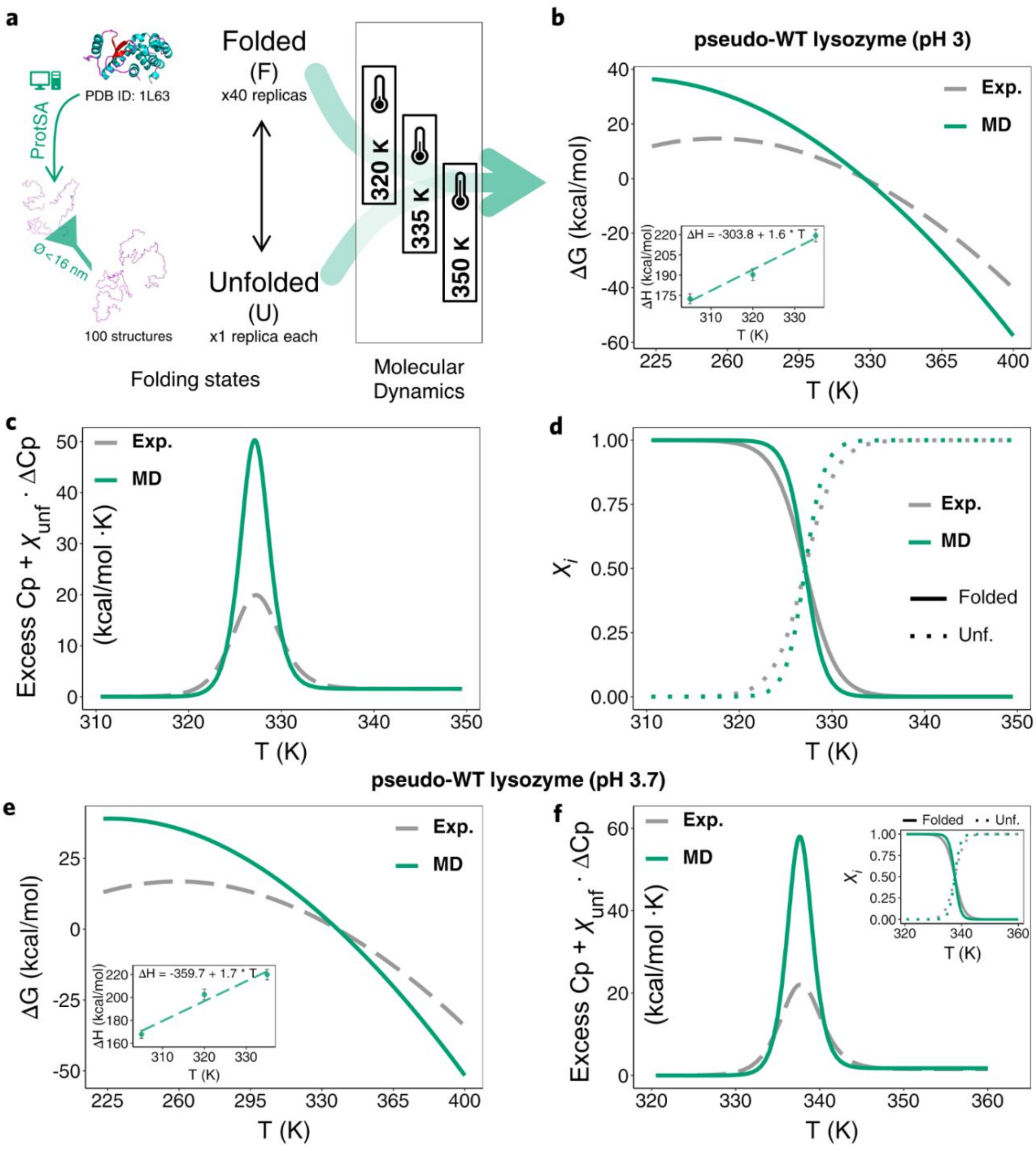

Figure S4. Simplified MD-based scheme and comparison with experimental results for pseudo-lysozyme. a) protein models and folding states, number of structures (unfolded) and replicas (folded) simulated, diameter cutoff used to filter too-elongated unfolded structures obtained from ProtSA[25] (left, see also **Figure S5**), and temperatures selected for the MD-based calculation (Charmm22-CMAP) of pseudo-WT lysozyme thermodynamics. **b-d)** Global stability curves ($\Delta G_{unf}(T)$), thermograms (Excess Cp + $\chi_{unf}\cdot\times$ ΔCp vs. T), and protein molar fractions (χ_i) vs. T (computed vs. experimental), respectively, obtained for pseudo-WT lysozyme at pH 3.0. Inset in **b** depicts the ΔH_{unf} vs. T linear plot with the fitted equation (the slope being ΔCp_{unf}) obtained from the MD simulations. **e** and **f)** Global stability curves ($\Delta G_{unf}(T)$) and thermograms (computed vs. experimental), respectively, obtained for pseudo-WT lysozyme at pH 3.7. Inset in **e** similar to that in **b**. The protein species fraction (χ_i) vs. T plot is included as an inset in **f**. The colour-coding is indicated in the legends of the panels.

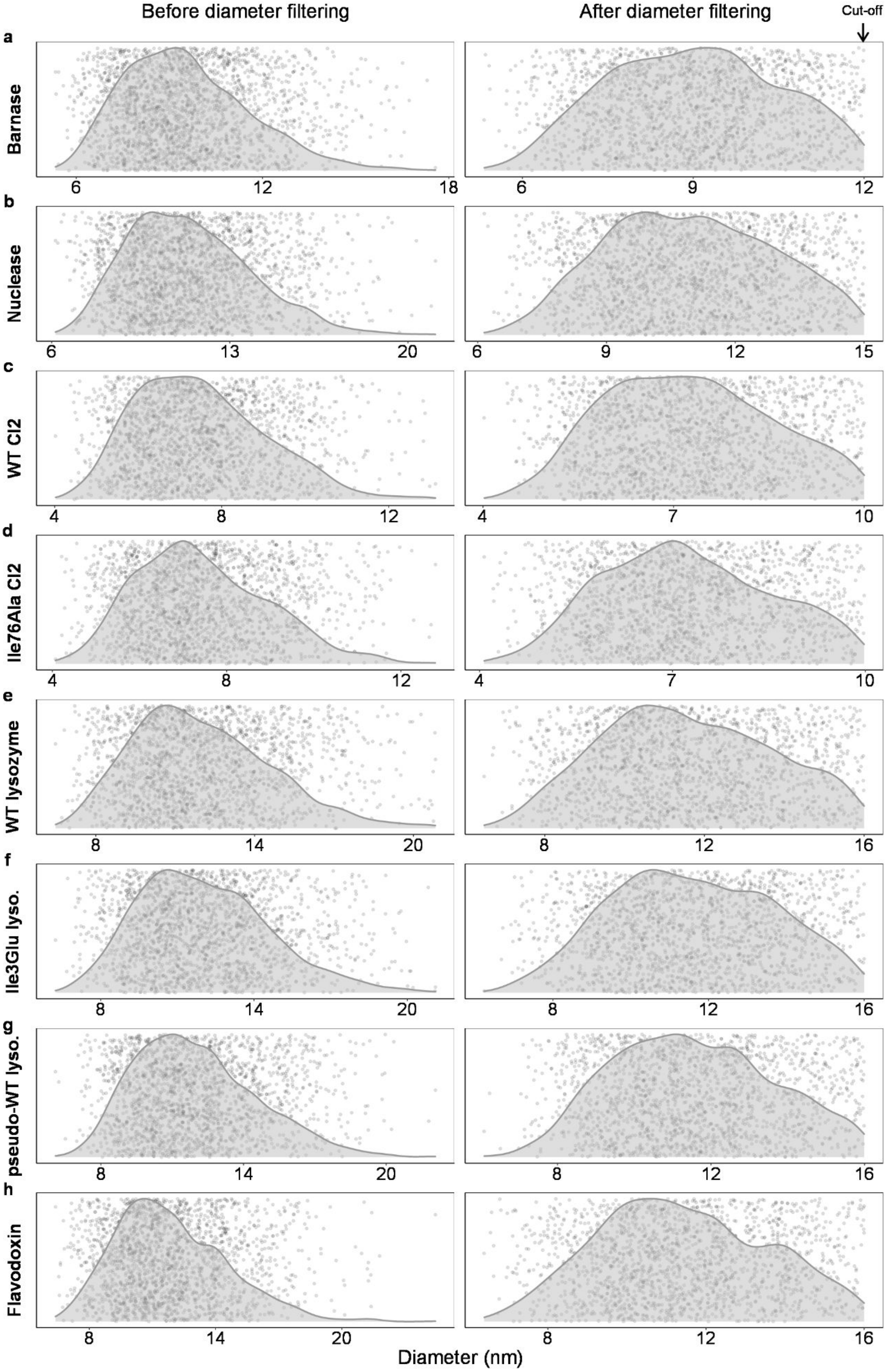
Before diameter filtering
After diameter filtering
Cut-off
a
Barnase
6
12
18
6
9
12
b
Nuclease
6
13
20
6
9
12
15
c
WT CI2
4
8
12
4
7
10
d
Ile76Ala CI2
4
8
12
4
7
10
e
WT lysozyme
8
14
20
8
12
16
f
Ile3Glu lyso.
8
14
20
8
12
16
g
pseudo-WT lyso.
8
14
20
8
12
16
h
Flavodoxin
8
14
20
8
12
16
Diameter (nm)

Figure S5. Diameter distributions and cutoffs applied to filter out too elongated structures in the unfolded ensembles generated by ProtSA. Scatter and density plots obtained before (left) and after (right) diameter filtering for **a)** Barnase, **b)** Nuclease **c)** WT CI2, **d)** Ile76Ala CI2) **e)** WT lysozyme, **f)** Ile3Glu lysozyme, **g)** pseudo-WT lysozyme and **h)** apoFld. Maximum diameter (applied cutoff) kept for the unfolded ensembles are the maximum values in the depicted *x*-axis scale in plots at the right-hand side (indicated in the top right-hand corner of the figure).

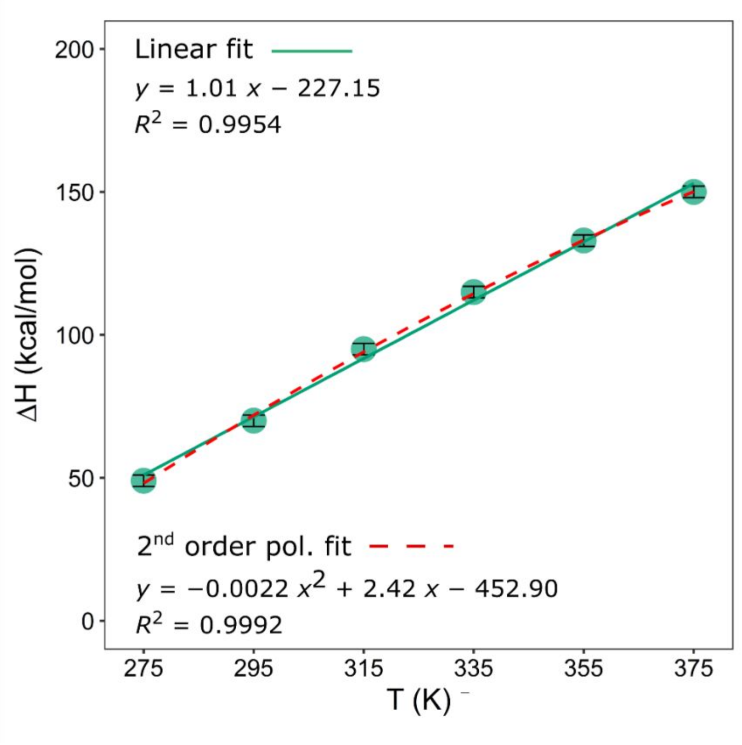

Figure S6. ΔH_{unf} vs. temperature plot obtained for barnase (pH 4.1, see Table S2) from six temperatures simulated over a range of 100 ºC. Linear and non-linear (2nd order polynomial) fittings are depicted. The corresponding equations and the squared Pearson correlation coefficients (R^2) are shown. The standard error of computed ΔH_{unf} at every temperature are indicated as error bars.

Supporting Information References

(1) Dolinsky, T. J.; Nielsen, J. E.; McCammon, J. A.; Baker, N. A. PDB2PQR: An Automated Pipeline for the Setup of Poisson-Boltzmann Electrostatics Calculations. *Nucleic Acids Res.* **2004**, *32* (WEB SERVER ISS.).

(2) Dolinsky, T. J.; Czodrowski, P.; Li, H.; Nielsen, J. E.; Jensen, J. H.; Klebe, G.; Baker, N. A. PDB2PQR: Expanding and Upgrading Automated Preparation of Biomolecular Structures for Molecular Simulations. *Nucleic Acids Res.* **2007**, *35* (SUPPL.2).

(3) Tan, Y. J.; Oliveberg, M.; Davis, B.; Fersht, A. R. Perturbed PKA-Values in the Denatured

States of Proteins. *J. Mol. Biol.* **1995**, *254* (5), 980–992.

(4) Van Der Spoel, D.; Lindahl, E.; Hess, B.; Groenhof, G.; Mark, A. E.; Berendsen, H. J. C. GROMACS: Fast, Flexible, and Free. *J. Comput. Chem.* **2005**, *26* (16), 1701–1718.

(5) Clark, A. F.; Hogg, R. W.; Gerken, T. A. Proton Nuclear Magnetic Resonance Spectroscopy and Ligand Binding Dynamics of the Escherichia Coli L-Arabinose Binding Protein. *Biochemistry* **1982**, *21* (9), 2227–2233.

(6) Fukada, H.; Sturtevant, J. M.; Quiocho, F. A. Thermodynamics of the Binding of L-Arabinose and of D-Galactose to the L-Arabinose-Binding Protein of Escherichia Coli. *J. Biol. Chem.* **1983**, *258* (21), 13193–13198.

(7) Søndergaard, C. R.; Olsson, M. H. M.; Rostkowski, M.; Jensen, J. H. Improved Treatment of Ligands and Coupling Effects in Empirical Calculation and Rationalization of PKa Values. *J. Chem. Theory Comput.* **2011**, *7* (7), 2284–2295.

(8) Carra, J. H.; Anderson, E. A.; Privalov, P. L. Thermodynamics of Staphylococcal Nuclease Denaturation. I. The Acid-denatured State. *Protein Sci.* **1994**, *3* (6), 944–951.

(9) Estrada, J.; Bernadó, P.; Blackledge, M.; Sancho, J. ProtSA: A Web Application for Calculating Sequence Specific Protein Solvent Accessibilities in the Unfolded Ensemble. *BMC Bioinformatics* **2009**, *10* (1), 104.

(10) Ayuso-Tejedor, S.; Angarica, V. E.; Bueno, M.; Campos, L. A.; Abián, O.; Bernadó, P.; Sancho, J.; Jiménez, M. A. Design and Structure of an Equilibrium Protein Folding Intermediate: A Hint into Dynamical Regions of Proteins. *J. Mol. Biol.* **2010**, *400* (4), 922–934.

(11) Galano-Frutos, J. J.; Sancho, J. Accurate Calculation of Barnase and SNase Folding Energetics Using Short Molecular Dynamics Simulations and an Atomistic Model of the Unfolded Ensemble: Evaluation of Force Fields and Water Models. *J. Chem. Inf. Model.* **2019**, *59* (10), 4350–4360.

(12) Robustelli, P.; Piana, S.; Shaw, D. E. Developing a Molecular Dynamics Force Field for Both Folded and Disordered Protein States. *Proc. Natl. Acad. Sci. U. S. A.* **2018**, *115* (21), E4758–E4766.

(13) Mackerell, A. D.; Feig, M.; Brooks, C. L. Extending the Treatment of Backbone Energetics in Protein Force Fields: Limitations of Gas-Phase Quantum Mechanics in Reproducing Protein Conformational Distributions in Molecular Dynamics Simulation. *J. Comput. Chem.* **2004**, *25* (11), 1400–1415.
